# Multimodal Lifestyle Intervention Improves Fatigue in Quiescent Inflammatory Bowel Disease: A Controlled Study

**DOI:** 10.1093/crocol/otaf009

**Published:** 2025-02-08

**Authors:** Roberta Loveikyte, Lola J M Koppelman, Mirjam J H Blijleven, Nathalie Wilmsen, Mar D M Rodríguez-Girondo, Sjaak Bloem, Philip W Voorneveld, Andrea E van der Meulen-de Jong, Sander van der Marel, P W Jeroen Maljaars

**Affiliations:** Department of Gastroenterology and Hepatology, Leiden University Medical Center, Leiden, the Netherlands; Department of Gastroenterology and Hepatology, University Medical Center Groningen, Groningen, the Netherlands; Department of Gastroenterology and Hepatology, Leiden University Medical Center, Leiden, the Netherlands; Voeding Leeft non-profit foundation, Amsterdam, the Netherlands; Voeding Leeft non-profit foundation, Amsterdam, the Netherlands; Department of Biomedical Data Sciences, Leiden University Medical Center, Leiden, the Netherlands; Center for Marketing & Supply Chain Management, Nyenrode Business University, Breukelen, the Netherlands; Department of Gastroenterology and Hepatology, Leiden University Medical Center, Leiden, the Netherlands; Department of Gastroenterology and Hepatology, Leiden University Medical Center, Leiden, the Netherlands; Department of Gastroenterology and Hepatology, Haaglanden Medical Center, The Hague, the Netherlands; Department of Gastroenterology and Hepatology, Leiden University Medical Center, Leiden, the Netherlands

**Keywords:** fatigue, inflammatory bowel disease, lifestyle intervention, nutrition

## Abstract

**Background:**

Lifestyle factors are significant contributors to fatigue, affecting ~45% of patients with inflammatory bowel disease (IBD). Hence, we evaluated the effect of a multimodal lifestyle intervention on fatigue in patients with IBD.

**Methods:**

Patients with quiescent IBD were enrolled in this multicenter, non-randomized, controlled interventional study. The intervention group followed a 12-month lifestyle program, which included digital group meetings with a nutritionist and a lifestyle coach focusing on nutrition, exercise, sleep, and relaxation. The program also encouraged patients to exercise more self-control over personal health. The control group received standard clinical care. Clinical data and patient-reported outcomes were collected. Fatigue was measured with the Functional Assessment of Chronic Illness Therapy-Fatigue (FACIT-F); any increase in FACIT-F was considered a positive response to the intervention. Inverse probability treatment weighting was used to correct confounding by indication.

**Results:**

Thirty-six patients in the intervention group and 32 in the control group were compared. More patients in the intervention group (82.1%) than in the control group (54.2%) experienced improvement in fatigue, *P* = .029, standardized mean difference (SMD) −0.624. Over 70% of patients in the intervention group achieved a clinically relevant improvement in fatigue. Compared to the control group, quality of life improved in the intervention group. Acceptance of the health status was a significant factor for fatigue improvement (*β* = 7.899, SE = 1.913, *P* < .001).

**Conclusions:**

Multimodal lifestyle intervention improves fatigue in patients with IBD. Acceptance appears essential for fatigue improvement; instruments evaluating acceptance could help to personalize treatment and maximize its effectiveness.

## Introduction

Fatigue is the most prevalent systemic symptom of inflammatory bowel disease (IBD)—a chronic immune-mediated condition characterized by relapsing-remitting inflammation of the gastrointestinal tract.^1,2^ Fatigue affects approximately 45% of patients with quiescent IBD and over 70% of patients with active disease.^3,4^ Fatigue reduces quality of life (QoL), hampers social life, household activities, and work productivity.^1,4–6^ It has been reported to be the foremost impediment in IBD, even more debilitating than gastrointestinal symptoms.^7–10^

The pathogenesis of fatigue is still elusive yet likely multifactorial. It has been established that poor sleep quality, sarcopenia, nutritional deficiencies leading to anemia (eg, iron deficiency) or medication-associated myelosuppression can reduce physical performance and lead to fatigue; association between fatigue and inflammation as well as intestinal dysbiosis has been widely reported, however, the underlying pathways are not well established.^1,11^ Similarly, there are no established guidelines focusing on the treatment of fatigue in IBD; nonetheless, treating potential reversible causes is advised in the latest European guidelines aimed at managing extraintestinal manifestations.^12^ Even after addressing these reversible and quantifiable causes—such as inflammation or anemia—few patients experience resolution in fatigue, emphasizing the need for novel interventions.^13^

Recently, an increasing number of studies focused on (chronic) fatigue. Psychological and lifestyle aspects, such as sleep disturbances, depression, or sarcopenia, have consistently been reported as major fatigue determinants in patients with IBD.^3,14,15^ Several non-pharmacologic interventions focusing on mindfulness or cognitive behavioral therapy have shown promising results in mitigating fatigue in IBD.^16–19^ In addition, interventions targeting nutrition and physical activity have reduced fatigue and improved QoL; however, controlled studies or head-to-head trials comparing different lifestyle interventions have not yet been done in the IBD population.^20–26^ Therefore, we investigated whether a multimodal lifestyle intervention can reduce fatigue in patients with quiescent IBD compared to routine clinical care according to the latest ECCO guidelines.

## Methods

### Study Population

Between February 2022 and March 2023, we recruited adult outpatients at the Leiden University Medical Center and the Haaglanden Medical Center in the Netherlands. Patients were eligible for inclusion in this multicenter, prospective, non-randomized, and controlled interventional study if they had an established IBD diagnosis—either Crohn’s Disease (CD), Ulcerative Colitis (UC), or IBD-unclassified (IBD-U)—and suffered from clinically relevant fatigue, defined as ≥4 and ≤8 on the Visual Analog Scale (VAS)^2^; patients with mild fatigue (fatigue VAS <4) or severe fatigue (fatigue VAS >8) were not eligible for inclusion because the severity of the complaints might limit their ability and motivation to participate in the study and adhere to the treatment modalities. Patients also had to be in remission, defined as fecal calprotectin (FCP) ≤150 mg/kg, in addition to at least three months of unaltered systemic pharmacotherapy. Patients in remission who were not receiving pharmacological maintenance treatment were also eligible for inclusion.

Patients were not eligible for inclusion if they were: severely underweight or overweight, defined as Body Mass Index <18.5 or >35 kg/m^2^; suffered from clinically relevant anemia (hemoglobin <7.0 mmol/L or <8.00 mmol/L for females and males, respectively) or nutritional deficiencies, that is, iron, vitamin D, vitamin B12, or folate deficiencies; had active infection or documented major surgery within 4 weeks prior to inclusion. Patients with the following comorbidities were also excluded: heart failure, liver cirrhosis, chronic obstructive pulmonary disease, inherited metabolic disorders, myelodysplastic syndrome, history of gastroduodenal surgery, end-stage renal disease (defined by an estimated glomerular filtration rate (eGFR) <30 mL/min/1.73m^2^), type 1 diabetes, type 2 diabetes treated with any other pharmaceutical agent than biguanides, or history of malignancy within the last 3 years. Dermatological non-melanoma malignancies were not considered an exclusion criterion. Pregnant or lactating women and patients with a history of psychiatric diseases, eating disorders, or addiction were also excluded. Patients with a history of depression were eligible only if they were in remission regarding the depression, defined as a Hospital Anxiety Depression Scale score <11 for the depression subscale.

### Study Intervention and Allocation

Participants were allocated to either the control or the intervention group. The allocation to a specific group was determined by the availability within the group and participants’ ability to attend the scheduled intervention. The control group received standard care that included an informational leaflet about the potential causes of fatigue in IBD and tips on how to manage it, which included advice to exercise more, eat a healthier diet, stop smoking, reduce alcohol intake, improve sleeping habits, and manage stress. Patients could ask for additional information or help as part of the routine care.

Patients in the intervention group followed a 12-month multimodal lifestyle intervention: an online lifestyle program provided by a non-profit organization Voeding Leeft that specializes in evidence-based lifestyle programs for different patient populations; a detailed description provided in the **Supplementary Table S11**.^25,27^ During the first 6 months, patients attended monthly plenary and small-group sessions guided by a nutritionist and a lifestyle coach. During the sessions, the Voeding Leeft team provided information and counseled patients about nutrition, physical activity, sleep, and relaxation. The program also encouraged participants to exercise more self-control over their health. The lifestyle program used the Integrated Change Model (ie, the I-Change Model, which states that behaviors are determined by the motivation or intention to carry out a particular behavior) to achieve a long-lasting behavioral change through 3 phases: awareness, motivation, and action.^28,29^ Alongside counseling and informative content, patients formulated personal goals and received daily tasks to facilitate gradual and sustainable goal attainment. Each month of the intervention centered around a specific lifestyle aspect; nevertheless, nutrition always played an important role. The nutritional advice aligned closely with the Mediterranean diet, emphasizing an unprocessed and plant-based diet enriched with small quantities of animal protein, such as fish or fermented dairy. The lifestyle program advised against frequent snacking and recommended to limit the number of mealtimes to 3 a day, including limiting alcohol consumption. Following the initial 6 months, attendance at online sessions was not mandatory; however, patients could attend online sessions every 6–12 weeks to stay motivated and foster the healthier habits they acquired throughout the course.

In addition to the group sessions, patients in the intervention group received access to an online platform containing additional background information, recipes, tips, guided breathing exercises, podcasts, and various physical exercises. The platform was accessible throughout the study period, enabling patients to monitor their progress and communicate with fellow participants and the Voeding Leeft team, including a nutritionist.

### Data Collection

Demographic, anthropometric, and clinical data, including Montreal classification (**Table 1** and **Supplementary Table S2**) were extracted from medical records at baseline.^30,31^ Clinical data were also extracted from medical records after 3 and 6 months of intervention. In addition, we collected patient-reported outcomes at baseline and after 3, 6, and 12 months. The instruments used in the study are described extensively in **Supplementary Table S1**. Fatigue was evaluated using fatigue VAS (higher scores indicating worse fatigue) and Functional Assessment of Chronic Illness Therapy-Fatigue (FACIT-F), with higher scores indicating better functioning.^32,33^ Health-related QoL (HRQoL) was assessed using the Short Inflammatory Bowel Disease Questionnaire (SIBDQ) and EuroQoL Five-dimensions and Five-levels (EQ-5D-5L); higher scores of both questionnaires reflect better HRQoL.^34,35^ Perceived Stress Scale (PSS) and Brief Illness Perception Questionnaire (B-IPQ) were used to evaluate perceived stress and the perception of IBD as a health threat, where higher scores indicate worse outcomes.^36–39^ The Health Monitor questionnaire was utilized to assess acceptance and perceived control, where ≥5 scores indicated high perceived control and acceptance of the disease and health status.^40,41^

**Table 1:** Baseline demographic and clinical characteristics of the study population, after inverse probability of treatment weighing.

	After the IPTW			
	Intervention	Control	*P*-value	SMD
Female sex (*n*, %)	17 (51.50%)	19 (51.40%)	.989	0.012
Age (years)	37.14 [31.64–48.32]	43.00 [30.20–50.00]	.906	0.052
BMI (kg/m^2^)	25.55 [22.61–28.60]	25.34 [22.53–28.98]	.802	-0.044
IBD disease duration (years)	14.19 [8.79–21.03]	11.00 [4.00–19.92]	.014	-0.396
IBD type: UC (*n*, %)	13 (38.20%)	13 (35.10%)	.786	-0.071
Fecal calprotectin (µg/g)	22.25 [10.00–51.50]	33.00 [13.91–43.51]	.624	-0.088
C-reactive protein (mg/L)	1.70 [0.81–4.00]	2.15 [1.00–3.36]	.494	0.216
Ulcerative colitis‡ (n, %)				
Age of onset (*n*, %)				
<17 years old	4 (28.60%)	3 (25.00%)		
17–40 years old	9 (64.30%)	7 (58.30%)		
>40 years old	1 (7.10%)	2 (16.70%)		
Disease extension (*n*, %)			.333	0.311
Pancolitis	3 (23.10%)	4 (30.80%)		
Left-sided colitis	8 (61.50%)	9 (69.20%)		
Proctitis	2 (15.40%)	0 (0.00%)		
Crohn’s disease (*n*, %)				
Age of onset (*n*, %)				
<17 years old	3 (15.00%)	6 (25.00%)		
17–40 years old	15 (75.00%)	13 (54.20%)		
>40 years old	2 (10.00%)	5 (20.80%)		
Disease location (*n*, %)			.244	0.478
Terminal ileum	11 (52.40%)	8 (33.30%)		
Colon	3 (14.30%)	2 (8.30%)		
Ileocolon	7 (33.30%)	14 (58.30%)		
Upper-GI disease*	3 (15.00%)	6 (25.00%)	.413	0.166
Perianal disease**	8 (40.00%)	5 (21.70%)	.193	-0.378
Disease behavior (*n*, %)			.099	-0.628
Inflammatory	12 (57.10%)	20 (83.30%)		
Stricturing	4 (19.00%)	3 (12.50%)		
Penetrating	5 (23.80%)	1 (4.20%)		
Medication (*n*, %)				
Aminosalicylates	10 (29.40%)	12 (32.40%)	.783	0.030
Immunomodulators	12 (35.30%)	11 (29.70%)	.617	-0.098
Biologics	21 (61.80%)	24 (64.90%)	.786	0.059
Small molecules	0 (0.00%)	1 (2.70%)	.334	0.198
Corticosteroids†	1 (2.90%)	4 (10.80%)	.195	0.310
History of surgery (*n*, %)				
(Procto)colectomy	1 (3.00%)	1 (2.70%)	.935	-0.114
Ileocoecal resection	6 (18.20%)	4 (10.80%)	.379	-0.217
History of smoking (*n*, %)			**<.001**	0.342
Never	21 (61.80%)	23 (62.20%)		
Current	0 (0.00%)	10 (27.00%)		
Former	13 (38.20%)	4 (10.80%)		
Educational status (*n*, %)			.563	0.104
Primary education	1 (2.90%)	0 (0.00%)		
Secondary education	16 (47.10%)	17 (45.90%)		
Higher education	17 (50.00%)	20 (54.10%)		

Initially, 40 participants were allocated to the intervention group and 32 to the control group; 4 participants dropped out at or just after baseline. Consequently, 36 participants in the intervention group and 32 in the control group were compared. For an overview of available and missing data per outcome measure refer to Supplementary Table S2. Data are presented as median [interquartile ranges] or as absolute numbers of available data (n) with corresponding percentages (%). BMI: body mass index; GI: gastrointestinal tract; IBD: Inflammatory Bowel Disease; IPTW: Inverse Probability of Treatment Weighing; SMD: Standardized Mean Difference; UC: ulcerative colitis. ‡Includes patients with ulcerative colitis and one patient with IBD-Unclassified. *Upper GI-involvement (Montreal L4) is presented as a modifier, which includes isolated upper GI-involvement and upper GI-involvement in addition to other disease locations. **Perianal disease (Montreal p) is presented as a modifier—indicating solely perianal disease involvement separate from penetrating disease behavior. †Systemic corticosteroids. Two-tailed *P*-values <.05 were considered statistically significant; the Benjamini–Hochberg procedure was used to adjust for multiple testing, adopting a 5% false discovery rate (FDR). *P*-values in **bold** indicate statistical significance after adjustment for multiple testing.

Different lifestyle aspects were also evaluated. The Pittsburgh Sleep Quality Index (PSQI) was used to assess sleep, with higher scores indicating worse sleep quality.^42,43^ An adjusted Mediterranean Diet Serving Score (adjusted MDSS) was used to evaluate adherence to the Mediterranean-like diet recommended during the lifestyle intervention, and the Short Questionnaire to Assess Health-enhancing physical activity (SQUASH) was used to assess physical activity levels.^44,45^ Moreover, the Work Productivity and Activity Impairment questionnaire (WPAI) was utilized to evaluate activity impairment, where higher scores indicate significant impairment; the iMTA Medical Consumption Questionnaire (iMCQ) was used to quantify healthcare consumption in local currency.^46–49^

### Sample Size and the Primary Outcome

Any increase in FACIT-F scores after 6 months of treatment was considered an improvement in fatigue. Based on previous studies, we estimated that 66% of patients in the intervention group would respond to the intervention compared with 33% in the control group.^25^ We aimed to include 35 patients in each treatment group to detect this difference with 80% power and a 5% significance level. We aimed to include 53 patients in each treatment group to account for 33% attrition, based on a previous study.^25^

### Statistical Analysis

All data, including the last measurement before the exclusion of a participant, were used in the analyses. Descriptive summaries are reported as means with standard deviations (mean ± SD) or medians with interquartile ranges [IQR, Q1–Q3] for continuous variables. Categorical variables are presented as absolute numbers of available data (*n)* with corresponding percentages (%). Normality assessment was performed by visually inspecting normal probability plots (*Q*–*Q*) and histograms. Mann–Whitney *U*-tests, chi-square, or independent sample *t*-tests were used to compare differences and changes (Δ) in patient-reported outcomes between independent (sub)groups. Spearman’s rank correlation coefficients (*ρ*) were used to determine correlations between continuous variables. Paired analyses using Wilcoxon’s rank sum test or paired *t*-tests were used to compare outcomes within individuals between different time points. In addition, the percentage of participants—who reached the Minimal Clinically Important Difference regarding fatigue (FACIT-F = 4), QoL (SIBDQ = 9, EQ VAS = 4.2), and activity impairment (WPAI = 7)—in the intervention and the control group after three and six months of treatment were compared using the chi-square test.^50,51^ Lastly, quality-adjusted life-years (QALYs) were calculated using the EQ-5D Index, measured with the EQ-5D-5L questionnaire.

Inverse probability treatment weighting (IPTW) was used to correct confounding by indication. Propensity scores were calculated to compare groups based on the probability of receiving the lifestyle intervention; the scores were calculated using multivariable binary logistic regression with pre-defined variables: sex, age, use of immunosuppressants (ie, thiopurines or methotrexate), Health Monitor: acceptance and perceived control scores, and baseline fatigue VAS. In addition, IBD disease duration was included in the binary logistic regression because a longer history of IBD disease might affect acceptance of the disease. A stabilized weight was calculated for every patient, accounting for the probability of exposure to the lifestyle intervention in the study population. After weighing, a between-group imbalance for the pre-defined variables was considered small if standardized mean differences ([SMD] estimated by Cohen’s *d*) <0.2 and moderate if SMD <0.5.^52^ Analyses for baseline differences, primary and secondary outcomes were repeated with the addition of stabilized weights. While attendance during the lifestyle intervention was monitored, participants who have missed a session could still access the information on the online platform; therefore, the attendance at the meetings was not included as a variable in the statistical analysis.

Furthermore, multivariable linear mixed-effects regression analyses were performed to assess the effect of treatment and changes in fatigue, QoL, sleep quality, disease perception, and perceived stress throughout the study period. Linear mixed-effects models were developed with an individual-specific random intercept and the following pre-defined individual covariates as fixed-effects: time, treatment group, age, sex, smoking status at baseline, weight at baseline, IBD type, education level, and Health Monitor scores for acceptance and perceived control, and an interaction between time and treatment group.

Two-tailed *P*-values <.05 were considered statistically significant; the Benjamini-Hochberg procedure was used to adjust for multiple testing given a relatively large number of outcomes, considering significance under a false discovery rate of 5%. Statistical analysis was performed with SPSS Statistics 29 software (SPSS Inc., Chicago, IL, USA). The proportion of available data for individual variables is presented in **Supplementary Table S2**; imputation methods for missing data were not utilized.

### Ethical Considerations

The study was approved by the Institutional Review Board at the Leiden University Medical Center (IRB no. P21.078) and the research institute at the participating center. All participants provided written informed consent. The study has been performed in accordance with the principles of the Declaration of Helsinki (2013) and has been prospectively registered in the ClinicalTrials.gov register: NCT05374967.

## Results

Seventy-two patients were initially allocated to the treatment groups. Four patients in the intervention group dropped out at or just after the baseline and were not included in the statistical analysis; consequently, 36 patients in the intervention group and 32 in the control group were compared. **Table 1** presents the baseline demographic, anthropometric, and clinical characteristics of the study population after performing the IPTW; results before the IPTW are presented in **Supplementary Table S3**. Sex, mean age, and educational level did not differ between groups. However, the control group contained more current smokers than the intervention group (**Table 1**).

Both groups had a similar number of patients with UC and CD, were in biochemical remission, represented by low median FCP and CRP levels, and did not differ regarding pharmacological therapy or history of surgery (**Table 1**). Patients in the intervention group had a longer IBD disease course (14.19 years [8.79–21.03 years]) compared with the control group (11.00 years [4.00–19.92 years]; *P* = .014. However, the difference was not statistically significant after adjusting for multiple testing.

After performing IPTW, we did not observe differences in patient-reported outcomes—such as fatigue, perceived stress and control, and sleep quality—between the intervention and the control groups. A comparison of patient-reported outcomes between the study groups before and after the IPTW is presented in **Supplementary Table S4**.

### Multimodal Lifestyle Intervention Improves Fatigue

After 6 months, more patients experienced an improvement in fatigue in the intervention (82.10%) than in the control group (54.20%), *P* = .029 (**Table 2**). This improvement was also greater in the intervention group compared with the control group: 12.5 points vs. a point increase in FACIT-F scores for the intervention and the control group, respectively. These significant differences were also observed after the IPTW (**Table 2**). In addition, more patients in the intervention group (71.40%) than in the control group (33.30%) experienced a Minimal Clinically Important Difference (MCID)—in other words, a clinically meaningful difference—regarding fatigue improvement, *P *= .006; however, this difference did not remain significant after correction for multiple testing and IPTW (**Table 4**).

**Table 2.** Number of patients who experienced a decrease in fatigue after six months of treatment, stratified by treatment type.

	Before the IPTW			
	Intervention	Control	*P*-value	SMD
Available data (*n*)	28	24	*NA*	*NA*
Increase in FACIT-F (*n*, %)	23 (82.10%)	13 (54.20%)	**.029**	−0.624
∆FACIT-F at 6 months	12.50 [5.96–21.75]	1.00 [−12.75 to 9.75]	**.002**	−1.078
	**After the IPTW**			
	**Intervention**	**Control**	***P*-value**	**SMD**
Increase in FACIT-F (*n*, %)	21 (84.00%)	16 (55.20%)	**.023**	−0.665
∆FACIT-F at 6 months	13.00 [6.23–21.38]	1.00 [−13.00 to 10.77]	**.001**	−1.054

Six months after the baseline, there were 32 participants in the intervention and 31 in the control group. For an overview of available and missing data per outcome measure refer to Supplementary Table S2. Data are presented as median [interquartile ranges] or as absolute numbers of available data (*n*) with corresponding percentages (%). IPTW: Inverse Probability of Treatment Weighing; FACIT-F: The Functional Assessment of Chronic Illness Therapy—Fatigue, lower scores indicate more severe fatigue; NA: not applicable; SMD: Standardized Mean Difference. ∆FACIT-F was calculated as the FACIT-F score after 6 months—FACIT-F score at baseline; a negative value indicates a decrease in FACIT-F scores. Two-tailed *P*-values <.05 were considered statistically significant; the Benjamini–Hochberg procedure was used to adjust for multiple testing, adopting a 5% false discovery rate (FDR). *P*-values in bold indicate statistical significance after adjustment for multiple testing.

**Table 4.** Minimal clinically important difference experienced by the study population after three or six months of intervention, stratified by treatment type.

	Before the IPTW				After the IPTW			
	Intervention	Control	*P*-value	SMD	Intervention	Control	*P-*value	SMD
After 3 months of intervention								
FACIT-F (*n*, %)	17 (54.80%)	7 (26.90%)	.033	−0.579	15 (50.00%)	11 (35.50%)	.252	−0.284
EQ-5D VAS (*n*, %)	17 (54.80%)	5 (19.20%)	**.006**	−0.772	15 (50.00%)	7 (22.60%)	.026	−0.555
SIBDQ (*n*, %)	5 (16.10%)	3 (11.50%)	.619	−0.130	5 (16.70%)	7 (22.60%)	.561	0.137
After 6 months of intervention								
FACIT-F (*n*, %)	20 (71.40%)	8 (33.30%)	**.006**	−0.810	18 (72.00%)	13 (43.30%)	.033	−0.647
EQ-5D VAS (*n*, %)	16 (57.10%)	6 (25.00%)	**.019**	−0.674	14 (56.00%)	7 (23.30%)	.013	−0.732
SIBDQ (*n*, %)	5 (17.90%)	3 (12.50%)	.594	−0.146	4 (16.70%)	6 (20.00%)	.754	0.050
WPAI (*n*, %)								
Presenteeism	11 (61.10%)	7 (43.80%)	.311	−0.343	11 (64.70%)	6 (37.50%)	.118	−0.516
Activity Impairment	16 (57.10%)	11 (47.80%)	.507	−0.184	14 (58.30%)	13 (44.80%)	.328	−0.270

Initially, 40 participants were allocated to the intervention group and 32 to the control group; four participants dropped out at or just after baseline. Consequently, 36 participants in the intervention group and 32 in the control group were compared. Three months after the baseline, there were 33 participants in the intervention group and 32 in the control group. Six months after the baseline, there were 32 participants in the intervention and 31 in the control group. For an overview of available and missing data per outcome measure refer to Supplementary Table S2. Data are presented as an absolute number of available data (*n*) with corresponding percentages (%) who experienced the minimal clinically important difference (MCID) in patient-reported outcomes. MCID represents the smallest change in a treatment outcome that an individual would identify as important. EQ-5D VAS: visual analog scale assesses the quality of life. FACIT-F: The Functional Assessment of Chronic Illness Therapy—Fatigue. IPTW: Inverse Probability of Treatment Weighing. SIBDQ: Short Inflammatory Bowel Disease Questionnaire assesses health-related quality of life. SMD: Standardized Mean Difference. WPAI: Work Productivity and Activity Impairment assesses work and activity; Presenteeism indicates impairment at work, whereas Activity impairment represents impairment during daily activities outside work, with higher scores indicating worse impairment. Two-tailed *P*-values <.05 were considered statistically significant; the Benjamini–Hochberg procedure was used to adjust for multiple testing, adopting a 5% false discovery rate (FDR). *P*-values in bold indicate statistical significance after adjustment for multiple testing.

The significant increase in the total FACIT-F scores in the intervention group was primarily driven by improvements in physical well-being (median 3-point increase, *P* < .001), functional well-being (median 1.5-point increase, *P* = 0.007), and the fatigue subscale (median 6-point increase, *P* < 0.001), as shown in **Table 3**. In contrast, median scores for these domains decreased or did not change in the control group. The same patterns were observed after the IPTW (**Table 3**).

**Table 3.** Changes and differences between treatment groups regarding patient-reported outcomes after 6 months of intervention.

	Before the inverse probability of treatment weighing									
	Intervention				Control					
	Baseline	After 6 months	*P*-value	SMD	Baseline	After 6 months	*P*-value	SMD	*P*-value for ∆	SMD for ∆
FACIT-F	98.00 [86.00–112.17]	111.50 [96.50–122.50]	**<.001**	−1.132	113.00 [96.00–124.00]	116.42 [86.50–133.25]	.966	0.045	**.002**	1.078
Physical Well-Being	21.00 [17.00–24.00]	24.00 [20.00–25.00]	**<.001**	−0.903	23.00 [19.00–24.00]	23.50 [18.00–25.00]	.706	0.023	**.007**	0.856
Social Well-Being	21.00 [17.00–22.00]	20.00 [16.08–22.13]	.710	−0.103	20.00 [18.00–23.00]	20.00 [18.25–24.75]	.128	−0.318	.573	−0.144
Emotional Well-Being	19.00 [16.00–20.00]	19.00 [15.00–20.00]	.602	−0.066	20.00 [18.00–22.00]	20.00 [15.75–21.75]	.578	0.151	.237	0.224
Functional Well-Being	16.00 [13.00–20.00]	17.50 [14.00–20.00]	**.007**	−0.591	20.00 [17.00–21.00]	19.50 [14.50–21.75]	.210	0.310	**.005**	0.878
Fatigue	26.00 [20.00–30.00]	32.00 [28.00–39.00]	**<.001**	−1.188	32.00 [22.00–39.00]	32.00 [17.75–43.00]	1.000	−0.012	**<.001**	1.142
EQ-5D VAS	63.00 [52.00–72.00]	63.50 [49.25–79.00]	.220	−0.216	75.00 [66.00–80.00]	70.00 [44.25–78.00]	.074	0.456	**.016**	0.689
B-IPQ	42.00 [32.00–47.00]	35.50 [30.00–43.50]	**.005**	0.603	34.00 [26.50–42.00]	30.00 [27.00–43.00]	.626	−0.105	**.016**	−0.767
Acceptance	3.33 [2.67–4.33]	3.67 [3.00–5.33]	**.001**	−0.777	4.33 [3.33–5.67]	4.67 [3.08–5.67]	.872	<0.001	**.020**	0.636
Perceived control	4.33 [3.67–5.00]	5.17 [4.00–5.92]	**<.001**	−0.839	4.67 [4.00–5.67]	4.67 [3.75–5.33]	.294	0.173	**<.001**	0.952
Adjusted MDSS	6.00 [5.00–7.00]	9.00 [8.00–10.00]	**<.001**	−1.554	6.00 [5.00–8.00]	8.00 [6.00–8.00]	.110	−0.335	**<.001**	1.394
	After the inverse probability of treatment weighing									
	**Intervention**				**Control**					
	**Baseline**	**After six months**	***P*-value**	**SMD**	**Baseline**	**After 6 months**	***P*-value**	**SMD**	***P*-value for ∆**	**SMD for ∆**
FACIT-F	106.46 [90.09–121.61]	115.66 [102.19–136.00]	**<.001**	−1.211	106.00 [88.00–116.86]	112.19 [83.00–130.00]	.872	0.041	**.001**	1.054
* Physical Well-Being*	21.00 [18.43–24.00]	24.00 [21.37–25.33]	**<.001**	−0.934	21.12 [16.44–24.00]	21.65 [18.00–25.00]	.534	0.086	**.004**	0.900
* Social Well-Being*	21.00 [17.00–23.09]	20.00 [16.72–22.14]	.710	−0.074	21.00 [18.00–23.23]	21.00 [19.84–25.80]	.032	−0.410	.360	−0.253
*Emotional Well-Being*	19.00 [16.80–20.00]	19.00 [15.00–20.00]	.521	−0.084	19.00 [15.00–21.00]	19.00 [14.00–21.00]	.913	0.040	.432	0.113
*Functional Well-Being*	17.86 [14.03–21.61]	18.00 [15.18–21.12]	**.006**	−0.642	18.74 [17.00–20.00]	17.78 [13.93–21.00]	.091	0.341	**.002**	0.911
* Fatigue*	27.00 [22.00–32.53]	35.08 [28.00–41.12]	**<.001**	−1.248	26.51 [16.00–33.91]	30.78 [10.00–40.00]	.775	−0.005	**<.001**	1.177
EQ-5D VAS	66.30 [60.00–74.95]	68.22 [54.39–80.00]	.202	−0.275	70.30 [52.64–80.00]	70.00 [40.46–74.00]	.049	0.421	**.014**	0.701
B-IPQ	40.00 [31.22–46.14]	35.00 [28.01–42.00]	**.003**	0.633	37.20 [28.07–48.00]	37.09 [28.00–56.00]	.197	−0.293	**.002**	−0.973
Acceptance	3.84 [2.77–4.96]	4.04 [3.28–5.33]	**<.001**	−0.770	3.34 [2.67–4.91]	3.97 [2.67–5.58]	.657	−0.127	.027	0.445
Perceived control	4.33 [3.81–5.24]	5.33 [4.19–6.00]	**<.001**	−0.886	4.33 [3.67–5.23]	4.33 [3.00–5.01]	.329	0.069	**<.001**	0.817
Adjusted MDSS	6.00 [5.00–7.00]	9.00 [8.00–10.00]	**<.001**	−1.490	6.00 [5.00–8.00]	8.00 [6.00–8.00]	.105	−0.411	**<.001**	1.350

Initially, 40 participants were allocated to the intervention group and 32 to the control group; four participants dropped out at or just after baseline. Consequently, 36 participants in the intervention group and 32 in the control group were compared. Six months after the baseline, there were 32 participants in the intervention and 31 in the control group. For an overview of available and missing data per outcome measure refer to Supplementary Table S2. Data are presented as median [interquartile ranges] or as absolute numbers of available data (n) with corresponding percentages (%). Adjusted MDSS: adjusted Mediterranean Diet Serving Score evaluates dietary habits and adherence to the Mediterranean diet, higher scores indicate better adherence. B-IPQ: the Brief Illness Perception Questionnaire assesses the cognitive and emotional representations of illness; higher scores indicate that a disease is perceived as a health threat. EQ-5D VAS: visual analog scale ranging from 0 (worst health) to 100 (best health). FACIT-F: The Functional Assessment of Chronic Illness Therapy—Fatigue, lower scores indicate more severe fatigue. SMD: Standardized Mean Difference. Two-tailed *P*-values <.05 were considered statistically significant; the Benjamini–Hochberg procedure was used to adjust for multiple testing, adopting a 5% false discovery rate (FDR). *P*-values in bold indicate statistical significance after adjustment for multiple testing. The *P*-value for ∆ indicates the statistical difference in changes between the treatment groups.

Although the effect on fatigue regressed between the end of the active intervention (ie, six months post-baseline) and the end of the study (ie, 12 months post-baseline), it remained significantly better at 12 months than at the baseline [FACIT-F 98.00 [86.00–112.17] at baseline vs. 110.00 [95.00–131.33] at 12 months, *P* = 0.004; as shown in **Table 5**).

**Table 5.** Changes and differences in patient-reported outcome measures after 12 months of intervention, stratified by treatment type.

	Before the inverse probability of treatment weighing									
	Intervention				Control					
	Baseline	After 12 months	*P*-value	SMD	Baseline	After 12 months	*P*-value	SMD	*P*-value for ∆	SMD for ∆
FACIT-F	98.00 [86.00–112.17]	110.00 [95.00–131.33]	**.004**	−0.632	113.00 [96.00–124.00]	118.00 [95.88–129.00]	0.626	−0.057	.026	0.669
Physical well-being	21.00 [17.00–24.00]	23.00 [19.00–25.00]	.019	−0.465	23.00 [19.00–24.00]	23.00 [20.00–25.75]	0.249	−0.140	.250	0.344
Social well-being	21.00 [17.00–22.00]	20.00 [16.00–23.33]	.567	−0.141	20.00 [18.00–23.00]	21.00 [19.79–23.75]	0.824	−0.027	.857	0.139
Emotional well-being	19.00 [16.00–20.00]	19.00 [17.00–21.00]	.613	−0.083	20.00 [18.00–22.00]	20.00 [18.00–21.00]	0.755	0.069	.606	0.152
Functional well-being	16.00 [13.00–20.00]	19.00 [15.00–22.00]	.027	−0.406	20.00 [17.00–21.00]	19.00 [16.00–21.00]	0.244	0.267	.011	0.679
Fatigue	26.00 [20.00–30.00]	33.00 [27.00–41.00]	**<.001**	−0.810	32.00 [22.00–39.00]	33.00 [26.00–42.25]	0.486	−0.173	**.009**	0.778
EQ-5D VAS	63.00 [52.00–72.00]	66.00 [50.00–80.00]	.354	−0.152	75.00 [66.00–80.00]	70.00 [51.25–80.00]	0.131	0.323	.093	0.415
B-IPQ	42.00 [32.00–47.00]	33.50 [28.00–39.75]	**<.001**	0.921	34.00 [26.50–42.00]	33.00 [26.00–44.00]	0.580	−0.101	**<.001**	−1.094
Acceptance	3.33 [2.67–4.33]	4.33 [3.33–5.67]	.041	−0.467	4.33 [3.33–5.67]	5.00 [3.75–5.92]	0.337	−0.173	.389	0.322
Perceived control	4.33 [3.67–5.00]	4.33 [4.00–5.67]	.039	−0.290	4.67 [4.00–5.67]	4.67 [3.75–5.58]	0.782	0.068	.060	0.371
Adjusted MDSS	6.00 [5.00–7.00]	9.00 [8.00–10.00]	**<.001**	−1.183	6.00 [5.00–8.00]	6.00 [5.00–7.00]	0.832	0.057	**<.001**	1.405
	After the inverse probability of treatment weighing									
	**Intervention**				**Control**					
	**Baseline**	**After 12 months**	***P*-value**	**SMD**	**Baseline**	**After 12 months**	***P*-value**	**SMD**	***P*-value for ∆**	**SMD for ∆**
FACIT-F	106.46 [90.09–121.61]	110.00 [106.56–131.55]	**.006**	−0.476	106.00 [88.00–116.86]	105.17 [89.53–125.00]	.286	−0.112	.041	0.458
* Physical well-being*	21.00 [18.43–24.00]	23.00 [19.00–25.00]	.040	−0.265	21.12 [16.44–24.00]	20.96 [19.00–25.17]	.058	−0.205	.594	0.098
* Social well-being*	21.00 [17.00–23.09]	20.00 [16.45–23.50]	.611	−0.104	21.00 [18.00–23.23]	21.00 [18.20–24.00]	.971	0.011	.788	0.127
*Emotional well-being*	19.00 [16.80–20.00]	19.44 [17.00–21.00]	.691	0.005	19.00 [15.00–21.00]	19.00 [14.00–20.59]	.615	0.058	.633	0.056
*Functional well-Being*	17.86 [14.03–21.61]	21.00 [15.00–22.00]	.023	−0.406	18.74 [17.00–20.00]	17.48 [13.82–21.00]	.119	0.363	**.003**	0.775
* Fatigue*	27.00 [22.00–32.53]	33.04 [27.09–41.97]	**.001**	−0.605	26.51 [16.00–33.91]	28.22 [21.11–37.04]	.137	−0.303	.033	0.484
EQ-5D VAS	66.30 [60.00–74.95]	68.27 [56.47–80.00]	.432	−0.088	70.30 [52.64–80.00]	66.94 [50.00–77.10]	.065	0.276	.094	0.332
B-IPQ	40.00 [31.22–46.14]	33.00 [28.00–38.00]	**<.001**	0.908	37.20 [28.07–48.00]	38.10 [27.00–44.58]	.555	−0.087	**<.001**	−1.055
Acceptance	3.84 [2.77–4.96]	4.73 [3.33–5.67]	.054	−0.418	3.34 [2.67–4.91]	4.33 [2.59–5.67]	.140	−0.248	.501	0.156
Perceived control	4.33 [3.81–5.24]	4.33 [4.00–5.92]	.027	−0.217	4.33 [3.67–5.23]	4.33 [3.63–5.33]	.783	−0.041	.052	0.187
Adjusted MDSS	6.00 [5.00–7.00]	9.00 [8.00–10.00]	**<.001**	−1.135	6.00 [5.00–8.00]	6.36 [5.96–8.00]	.945	−0.022	**<.001**	1.315

Initially, 40 participants were allocated to the intervention group and 32 to the control group; at or just after baseline four participants dropped out. Consequently, 36 participants in the intervention group and 32 in the control group were compared. Six months after the baseline, there were 32 participants in the intervention and 31 in the control group. For an overview of available and missing data per outcome measure refer to Supplementary Table S2. Data are presented as median [interquartile ranges] or as absolute numbers of available data (n) with corresponding percentages (%). Adjusted MDSS: adjusted Mediterranean Diet Serving Score evaluates dietary habits and adherence to the Mediterranean diet, higher scores indicate better adherence. B-IPQ: the Brief Illness Perception Questionnaire assesses the cognitive and emotional representations of illness; higher scores indicate that a disease is perceived as a health threat. EQ-5D VAS: visual analog scale ranging from 0 (worst health) to 100 (best health). FACIT-F: The Functional Assessment of Chronic Illness Therapy—Fatigue, lower scores indicate more severe fatigue. SMD: Standardized Mean Difference. Two-tailed *P*-values <.05 were considered statistically significant; the Benjamini–Hochberg procedure was used to adjust for multiple testing, adopting a 5% false discovery rate (FDR). *P*-values in bold indicate statistical significance after adjustment for multiple testing. The *P*-value for ∆ indicates the statistical difference in changes between the treatment groups.

### Multimodal Lifestyle Intervention has a Positive Impact on Quality of Life

After six months, average QoL—measured by EQ-5D VAS—improved in the intervention group, whereas it decreased slightly in the control group (**Table 3**, **Supplementary Table S6**). In addition, more than 50% of patients in the intervention group achieved a MCID in EQ-5D VAS as early as 3 months after the baseline compared with only 19.20% of patients in the control group (*P* = .006); however, this result did not remain statistically significant after the IPTW and adjustment for multiple testing (**Table 4**). The same pattern was observed for QoL measures regarding the MCID after 6 months of treatment: a numerically larger proportion of patients experienced improvement in the intervention than the control group (**Table 4**).

While QoL stagnated in the control group, statistically non-significant improvements in QoL measures persisted in the intervention group at the end of the study (**Table 5**, **Supplementary Table S7**). In addition, patients in the intervention group gained 0.05 more QALYs throughout the study than patients in the control group. The QALYs at the end of the study were 0.850 for the intervention and 0.790 for the control group. In short, multimodal lifestyle intervention improved QoL and led to a greater gain in QALYs than routine care.

### Multimodal Lifestyle Intervention Improves Diet Quality But Not Other Lifestyle Factors

The difference in changes between the control and the intervention groups regarding lifestyle factors after six months of treatment are presented in **Table 3** and **Supplementary Table S6**. Sleep quality and perceived stress did not differ between the groups; however, we observed a significant improvement in diet quality in the intervention group, reflected by an increase in adjusted MDSS scores compared with the control group (*P* < 0.001). The diet quality improved as early as three months post-baseline (**Supplementary Table S5**) in the intervention group, whereas it remained unchanged in the control group.

At the end of the study, most patients (77.80%) in the intervention group said to follow the lifestyle advice somewhat; 22.20% of patients were no longer following the advice. The most common reasons for the suboptimal compliance were lack of time and difficulty adhering to the advice, for example, limiting sweets. The diet quality in the intervention group remained significantly better at the end of the study compared with the baseline and the control group (**Table 5**). In short, multimodal lifestyle intervention leads to a sustained improvement in diet quality.

### Multimodal Lifestyle Intervention Improves Disease Perception, Acceptance, and Control

An improvement in disease perception was observed after six months, reflected by decreased B-IPQ scores (**Table 3**). B-IPQ scores decreased more in the intervention group (42.00 [32.00–47.00] vs. 35.50 [30.00–43.50]; *P* = .005) than the control group (34.00 [26.50–42.00] vs. 30.00 [27.00–43.00]; *P* = .626). These changes were significantly different when comparing the two groups (*P *= .016), as presented in **Table 3**. Disease perception continued improving throughout the study in the intervention group but not in the control group (**Table 5**). In addition, acceptance of the health status and perceived control improved significantly in the intervention group after six months; this increase was also statistically significant compared with the control group (**Table 3**). In short, multimodal lifestyle intervention improved disease perception, acceptance of the health status, and perceived control.

### Factors Associated With Changes in Fatigue and Quality of Life

Improvement in FACIT-F scores over the course of the study were significantly correlated with positive changes in all other patient-reported outcomes except for changes in sleep, that is, PSQI scores (**Supplementary table S9**).

Multivariable linear mixed-effects regression analyses were performed to identify major factors influencing FACIT-F scores. We observed a statistically significant (*P* = 0.007) effect of an interaction between time and treatment group regarding changes in fatigue with an evident increase in FACIT-F scores over the course of the study in the intervention group compared with the control group, where scores remain constant. In addition, three factors were significantly associated with changes in fatigue, as detailed in **Table 6**. Increasing acceptance was associated with increasing FACIT-F scores throughout the study period, reflecting less fatigue (*β* = 7.899, SE = 1.913, *P* < .001). Time in treatment, with the greatest improvement occurring during the first 3 months of the intervention, and a college or university degree were associated with a reduction in fatigue in response to the treatment.

**Table 6.** Explanatory factors and predictors of fatigue and quality of life in patients with inflammatory bowel disease.

	Fatigue: FACIT-F				
	*β*	SE *β*	*t*-Value	*P*-value	95% CI
Intercept	46.045	18.758	2.455	.017	[8.514 to 83.577]
Treatment *(reference: control group)*	−0.176	5.776	−0.030	.976	[−11.651 to 11.300]
Sex *(reference: male)*	−6.501	4.567	−1.423	.160	[−15.655 to 2.653]
Age	0.218	0.197	1.107	.273	[−0.176 to 0.612]
Bodyweight	0.240	0.175	1.370	.176	[−0.111 to 0.592]
IBD type (*reference: UC*)	3.404	4.610	0.738	.463	[−5.838 to 12.646]
Never smoked *(reference: current smoker)*	−2.638	8.445	−0.312	.756	[−19.564 to 14.289]
Former smoker *(reference: current smoker)*	−10.625	9.454	−1.124	.266	[−29.575 to 8.325]
Primary education *(reference: higher education)*	1.931	18.103	0.107	.915	[−34.406 to 38.268]
Secondary education *(reference: higher education)*	−12.412	4.492	−2.763	.008	[−21.417 to −3.407]
Health monitor: acceptance	7.899	1.913	4.130	<.001	[4.066 to 11.732]
Health monitor: control	2.128	2.138	0.996	.324	[−2.161 to 6.417]
Time	0.413	0.901	0.458	.648	[−1.367 to 2.192]
Time * Intervention group *(reference: control group)*	3.390	1.233	2.749	.007	[0.955 to 5.825]
	**Quality of life: EQ-5D Index**				
	**β**	**SE β**	***t*-value**	***P*-value**	**95% CI**
Intercept	0.527	0.136	3.869	<.001	[0.254 to 0.799]
Treatment *(reference: control group)*	0.066	0.046	1.459	.147	[−0.024 to 0.157]
Sex *(reference: male)*	−0.061	0.033	−1.857	.069	[−0.126 to 0.005]
Age	0.001	0.001	0.462	.646	[−-0.002 to 0.003]
Bodyweight	0.003	0.001	2.000	.051	[−0.00001 to 0.005]
IBD type (reference: UC)	0.021	0.033	0.646	.521	[−0.045 to 0.087]
Never smoked *(reference: current smoker)*	−0.109	0.060	−1.810	.076	[−0.230 to 0.012]
Former smoker *(reference: current smoker)*	−0.178	0.067	−2.644	.011	[−0.314 to -0.043]
Primary education *(reference: higher education)*	−0.004	0.128	-0.030	.976	[−0.261 to 0.253]
Secondary education *(reference: higher education)*	−0.065	0.032	−2.028	.048	[−0.129 to −0.001]
Health monitor: acceptance	0.043	0.014	3.176	.003	[0.016 to 0.071]
Health monitor: control	−0.005	0.015	−0.317	.752	[−0.035 to 0.026]
Time	0.006	0.009	0.639	.524	[−0.012 to 0.023]
Time * intervention group *(reference: control group)*	−0.0005	0.012	−0.039	.969	[−0.024 to 0.024]

IBD: inflammatory bowel disease. Fatigue was measured by the Functional Assessment of Chronic Illness Therapy—Fatigue (FACIT-F), with lower scores indicating more severe fatigue. Quality of life was measured by the EQ-5D index, which represents health utility ranging from 0.0 (dead) to 1.0 (perfect health).

Similarly, improvements in QoL were greater in those with higher acceptance scores at baseline (β = 0.043, SE = 0.014, *P* = .003) and patients with a college or university degree (*P *= .048), as shown in **Table 6**. However, we did not observe a statistically significant effect (*P* = .969) of an interaction between time and treatment group regarding QoL. Similar patterns were observed for sleep quality, perceived stress, and disease perception with either time in treatment and/or acceptance and control as the most significant factors (**Supplementary Table S10**). In short, acceptance plays a significant role in achieving better outcomes, such as fatigue and QoL.

### Adverse Effects

In total, 66 adverse events were observed in the intervention group and 44 in the control group. All observed adverse events, including IBD flares, are presented in **Supplementary Table S8**. Most of the adverse events in both groups were unrelated to the study, including serious adverse events such as hospitalizations. The most common events were gastrointestinal complaints. A similar number of patients in both groups experienced an IBD flare during the study: nine patients in the intervention group and seven patients in the control group. Similarly, a comparable number of gastrointestinal complaints unrelated to IBD activity were observed in the intervention (*n* = 20) and the control group (*n* = 21). In short, the multimodal lifestyle intervention appears not to increase the risk of adverse events.

## Discussion

In this study, we evaluated whether a multimodal lifestyle intervention could improve fatigue in patients with IBD compared to routine care. Fatigue improved in 82% of patients in the intervention group compared with 54% in the control group at 6 months, and the difference between these groups remained significant at 12 months. The reduction in fatigue was clinically meaningful, given that most patients in the intervention group achieved a clinically relevant improvement in fatigue, suggesting multimodal lifestyle interventions could be used in routine care for fatigue management.

Improvements in fatigue might not always lead to better QoL. In other patient populations, improved fatigue has been reported to lead to better QoL, general well-being and daily functioning.^53,54^ Despite a significant reduction in fatigue during this study, we observed non-significant improvements in QoL measures in the intervention group compared with the control group. Limited existing data on lifestyle interventions in patients with IBD make the comparison with our results difficult. In a Dutch study, significant improvements in fatigue and the impact of disease on daily life (measured using the Inflammatory Bowel Disease Disability Index) were reported, yet health-related QoL remained unchanged after 6 months of combined intervention focusing on diet quality and physical activity.^24^ In contrast, our group has previously performed a pilot study evaluating the effect of the Mediterranean diet in patients with quiescent disease and observed significant improvements in fatigue and QoL.^25^ The patient population in the current study and the pilot study were similar, except patients in the current study were required to have clinically relevant fatigue. Patients participating in the current study might have been more fatigued and the improvements in fatigue might not have been significant enough to influence QoL to a more considerable extent. Nevertheless, patients in the intervention group gained 0.05 more QALYs throughout the study compared with the control group, suggesting lifestyle intervention positively influences QoL.

Opting for multimodal lifestyle interventions may be more advantageous than addressing singular lifestyle factors. The multifaceted nature of the intervention makes it difficult to pinpoint which factor or combination of factors was responsible for the reduction in fatigue; however, we observed a significant improvement in diet quality within the intervention group compared with the control group, indicating that dietary improvements may play a role in reducing fatigue. Additionally, improvements in disease perception, acceptance, and perceived control likely contributed to fatigue reduction. Whether a lack of statistically significant changes in stress and sleep scores prevent further or more significant improvements in fatigue is not clear, especially since measuring adherence to the advice given for sleep and stress improvement is difficult. In short, multimodal lifestyle interventions should be recommended for patients suffering from fatigue. Nutrition and acceptance of health status should be core components of these interventions, complemented with modules tailored to individual patient needs, such as stress management.

Acceptance plays an essential role in fatigue and QoL improvement. We postulate that patients’ attitudes towards their disease and the prescribed treatment might be a key factor influencing the efficacy of the intervention, given that not all patients in the intervention group showed improvements in fatigue and QoL. For example, difficulty accepting personal health status could lead to more severe fatigue due to the efforts spent actively fighting or denying the presence of a disease or specific symptoms such as fatigue. Likewise, we speculate that the perception of having no or little control over personal health could make patients passive and not seek a solution or a treatment, especially where personal efforts would be necessary. For optimal intervention outcomes, the Subjective Health Experience (SHE) model—that describes how subjective personal health drives health-related behavior including therapy compliance and self-management—advocates categorizing patients into four segments based on perceived control and acceptance scores, assuming patients in different segments need different types of interventions.^40,41,55,56^ In this study, 74.3% of patients in the intervention group had both low acceptance and control scores, suggesting that the counseling and guidance provided during the lifestyle intervention was the optimal intervention, as has been suggested by the SHE model. Patients with low acceptance and control scores require more personal guidance in learning that patient’s choices can and do influence their own health despite having and accepting the presence of a chronic disease.^40,41,56^ In contrast, patients with higher acceptance and/or control scores in the intervention group might have benefited from a different type of intervention, since this type of patients are more likely to seek information that could aid their quest for self-management rather than an intensive process of coaching or personal guidance, as has been proposed by the SHE model.^40,41,56^ Likewise, we observed a positive effect of routine clinical care on patients in the control group, which is unsurprising given that a quarter of these patients scored high in acceptance and control, indicating they benefit more from high-quality information than personal counseling. In summary, incorporating instruments to assess patient characteristics could personalize treatment and optimize improvements in fatigue, QoL, and other outcomes.

To the best of our knowledge, this is the first controlled study to evaluate the effect of a multimodal lifestyle intervention on fatigue in patients with IBD. However, the study has several limitations. First, we did not reach the pre-defined sample size. The recruitment was prolonged and challenging due to patients’ unwillingness to participate in the study. Consequently, the study population might represent more motivated patients, and the results might not be generalizable to the entire IBD population. In addition, there were more patients who smoke in the control group than the intervention group, while there were more patients who quit smoking sometime prior to inclusion in the intervention group. We postulate that smoking itself may not significantly affect chronic fatigue in patients with IBD, but it may reflect the patients’ motivation to change their habits, which could have influenced the fatigue outcomes in this study. Secondly, the relatively small sample size and recruitment difficulties made it unfeasible to incorporate randomization into this study, but we used IPTW to correct for confounding by indication and mitigate the absence of randomization. Thirdly, we did not include data on economic status, major life events (eg, death of a family member or divorce), or questionnaires focused on psychosocial aspects such as depression throughout the study. These data could have provided more information about why some patients experienced a reduction or improvement in fatigue and QoL. Lastly, an extensive number of questionnaires were used in this study, which might have affected the data quality. We observed that 2 of the longest questionnaires and also the last ones to be filled out—the iMCQ and the SQUASH—were often not filled out or filled out incorrectly; as a result, we were unable to score these questionnaires appropriately and likely have not observed the true effects of the treatment regarding healthcare consumption and physical activity.

Despite the limitations, we found that the multimodal lifestyle intervention improved fatigue, QoL, and disease perception in patients with IBD, which confirms previous findings from non-controlled studies.^22–24^ Multimodal lifestyle interventions should be recommended in daily practice for patients suffering from fatigue. Lifestyle interventions must be modular, meaning that nutrition and disease acceptance must be the main focus points, but auxiliary modules should be tailored to individual patient needs, such as sleep quality or anxiety. Utilizing instruments evaluating acceptance could further personalize treatment and maximize its effectiveness.

Fawson et al. have recently reported patients’ wishes for more information, online tools, and interventions, yet previous studies employing mindfulness or lifestyle interventions for fatigue in IBD have struggled with recruitment.^57,58^ Difficulties with recruitment during this and other studies evaluating lifestyle interventions should be addressed in prospective and qualitative studies, including patient preference for cognitive behavioral therapy.^59^ During this study, we have observed a slight regression in the improvement of fatigue after the initial six months; we speculate that decreasing adherence, motivation, and the difficulty sustaining the required effort for further improvement might have caused this. Therefore, future studies should aim to optimize the practicality of the lifestyle interventions—i.e., timing, the number and location of the sessions—to ensure patient compliance, program feasibility and cost-effectiveness. Until an objective measure or an instrument is available to quantify fatigue or quality of life, future studies should also attempt to control for potential Hawthorne effect, including objective measures of different lifestyle aspects related to fatigue (eg, measuring handgrip strength) next to patient-reported outcomes. Lastly, the discrepancy between patients’ wishes for more interventions and lack of participation should be evaluated, focusing on patients’ expectations, willingness to change their habits, and the therapeutic utility of tools regarding acceptance and perceived control.

## Conclusion

Multimodal lifestyle intervention improves fatigue and quality of life in patients with IBD. Acceptance appears essential for fatigue improvement; instruments evaluating acceptance could help to personalize treatment and maximize its effectiveness.

## Supplementary Material

otaf009_suppl_Supplementary_Tables

## Data Availability

The datasets used in this study are available upon reasonable request.
