# Supplementary material for: Multimodal Lifestyle Intervention Improves Fatigue in Quiescent Inflammatory Bowel Disease: A Controlled Study
Source: Crohns Colitis 360. 2025 Feb 8;7(1):otaf009. doi: 10.1093/crocol/otaf009 (PMC11906969; doi:10.1093/crocol/otaf009)
Supplement: otaf009_suppl_Supplementary_Tables [file otaf009_suppl_supplementary_tables.pdf]

SUPPLEMENTARY TABLES

Supplementary table 1: Instruments used to evaluate patient-reported outcomes.

Instrument	Items	Domains	Response	Total score	Interpretation
Fatigue VAS	1	Fatigue	VAS 0–10	0–10	A higher score indicates worse fatigue
FACIT-F[1]	40	Physical well-being<br>Social well-being<br>Emotional well-being<br>Functional well-being<br>Fatigue	5-point Likert-type scale	0–160	A higher score indicates a better outcome
SIBDQ[1]	10	Social<br>Bowel<br>Emotional<br>Systemic	7-point Likert-type scale	10–70	A higher score indicates better HRQoL
EQ-5D-5L[2]	6	Mobility<br>Self-care<br>Usual activities<br>Pain/discomfort<br>Anxiety/depression<br>EQ VAS	5-point Likert-type scale for the five domains<br><br>0–100 scale for EQ VAS	EQ Index 0–1<br>EQ VAS 0–100	A higher score indicates better HRQoL
B-IPQ[3]	9	Cognitive illness representation<br>Emotional illness representation<br>Illness comprehensibility<br>Causal representation	0–10 scale except for Causal representation, which is an open-ended question. It indicates the three most important factors causing the illness	0–80	A higher score indicates that a disease is perceived as threatening
PSS[4]	10	Perceived stress	5-point Likert-type scale	0–40	A higher score indicates worse perceived stress

[1] Tinsley A, Macklin EA, Korzenik JR, et al. Validation of the functional assessment of chronic illness therapy-fatigue (FACIT-F) in patients with inflammatory bowel disease. Aliment Pharmacol Ther 2011;34:1328-36.

[2] Irvine EJ, Zhou Q, Thompson AK. The Short Inflammatory Bowel Disease Questionnaire: a quality of life instrument for community physicians managing inflammatory bowel disease. CCRPT Investigators. Canadian Crohn's Relapse Prevention Trial. Am J Gastroenterol 1996;91:1571-8.

[3] Herdman M, Gudex C, Lloyd A, et al. Development and preliminary testing of the new five-level version of EQ-5D (EQ-5D-5L). Qual Life Res 2011;20:1727-36.

[4] de Raaij EJ, Schröder C, Maissan FJ, et al. Cross-cultural adaptation and measurement properties of the Brief Illness Perception Questionnaire-Dutch Language Version. Man Ther 2012;17:330-5.

WPAI[5]	6	Absenteeism<br>Presenteeism<br>Work productivity loss<br>Activity impairment	Number of hours (not-)worked.<br>A 10-point Likert-type scale also indicates how much IBD symptoms impair work and daily activities	0–100	A higher score indicates worse impairment
PSQI[6]	19	Subjective sleep quality<br>Sleep latency<br>Sleep duration<br>Sleep efficiency<br>Sleep disturbance<br>Use of sleep medication<br>Daytime dysfunction	4-point Likert-type scale<br><br>Bedtime and sleep duration in minutes or hours	0–21	A higher score indicates worse sleep quality
Adjusted MDSS[7]	17	Adherence to the Mediterranean diet	Units of consumed food	0–17	A higher score indicates better adherence to the Mediterranean diet
Health Monitor[8]	6	Acceptance<br>Perceived control	7-point Likert-type scale	≥5 or <5	≥5 score indicates high perceived control and acceptance of the disease and health status
iMCQ[9]	18	Primary care<br>Secondary care<br>Paramedic care<br>Institutional care<br>Homecare<br>Medication	Various response types inventorying healthcare consumption	Number (€)	A higher number indicates higher healthcare costs
SQUASH[10]	22	Commuting<br>Physical activity at work/school<br>Physical activity at home<br>Leisure physical activity	Number of days, hours, and minutes of physical activity	Min/week	Minutes of weekly light, moderate, and strenuous physical activity

Adjusted MDSS: adjusted Mediterranean Diet Serving Score; B-IPQ: Brief Illness Perception Questionnaire; EQ-5D-5L: EuroQoL Five-dimensions and Five-levels (EQ-5D-5L); FACIT-F: Functional Assessment of Chronic Illness Therapy – Fatigue; HRQoL: health-related quality of life; iMCQ: the iMTA Medical Consumption Questionnaire; PSS: Perceived Stress Scale; PSQI: the Pittsburgh Sleep Quality Index; SIBDQ: Short Inflammatory Bowel Disease Questionnaire; SQUASH: Short Questionnaire to Assess Health-enhancing physical activity; VAS: visual analog scale; WPAI: Work Productivity and Activity Impairment questionnaire.

5 Sexton KA, Walker JR, Graff LA, et al. Evidence of Bidirectional Associations Between Perceived Stress and Symptom Activity: A Prospective Longitudinal Investigation in Inflammatory Bowel Disease. Inflammatory Bowel Diseases 2017;23:473-483.

6 Sandborn W, Reilly M, Brown M, et al. P-046: Determination of the minimally important difference in WPAI: CD score that indicates a relevant impact on work productivity. Inflammatory Bowel Diseases 2008;14:S24-S24.

7 Buysse DJ, Reynolds CF, 3rd, Monk TH, et al. The Pittsburgh Sleep Quality Index: a new instrument for psychiatric practice and research. Psychiatry Res 1989;28:193-213.

8 Martínez-González MA, García-Arellano A, Toledo E, et al. A 14-item Mediterranean diet assessment tool and obesity indexes among high-risk subjects: the PREDIMED trial. PLoS One 2012;7:e43134.

9 Bloem S, Stalpers J, Groenland EAG, et al. Segmentation of health-care consumers: psychological determinants of subjective health and other person-related variables. BMC Health Serv Res 2020;20:726.

10 Kanters TA, Bouwmans CAM, van der Linden N, et al. Update of the Dutch manual for costing studies in health care. PLoS One 2017;12:e0187477.

11 Wendel-Vos GC, Schuit AJ, Saris WH, et al. Reproducibility and relative validity of the short questionnaire to assess health-enhancing physical activity. J Clin Epidemiol 2003;56:1163-9.

Supplementary table 2: Overview of the percentage of available data for individual variables.

	**All (*n* = 68)**	**Intervention (*n* = 36)**	**Control (*n* = 32)**
Sex (n, %)	68 (100%)	36 (100%)	32 (100%)
Age (years)	68 (100%)	36 (100%)	32 (100%)
BMI (kg/m^2)	68 (100%)	36 (100%)	32 (100%)
IBD disease duration (years)	68 (100%)	36 (100%)	32(100%)
IBD type	68 (100%)	36 (100%)	32 (100%)
Fecal calprotectin	67 (98.53%)	35 (97.22%)	32 (100%)
C-reactive protein	66 (97.06%)	36 (100%)	30 (93.75%)
Ulcerative colitis‡ (n, %)	*n* = 25	*n* = 14	*n* = 11
Age of onset (n, %)	25 (100%)	14 (100%)	11 (100%)
Disease extension (n, %)	25 (100%)	14 (100%)	11 (100%)
Crohn's disease (n, %)	*n* = 43	*n* = 22	*n* = 21
Age of onset (n, %)	43 (100%)	22 (100%)	21 (100%)
Disease location (n, %)	43 (100%)	22 (100%)	21 (100%)
*Upper-GI disease**	43 (100%)	22 (100%)	21 (100%)
*Perianal disease***	43 (100%)	22 (100%)	21(100%)
Disease behavior (n, %)	43 (100%)	22 (100%)	21 (100%)
Medication (n, %)			
Aminosalicylates	68 (100%)	36 (100%)	32 (100%)
Immunomodulators	68 (100%)	36 (100%)	32 (100%)
Biologics	68 (100%)	36 (100%)	32 (100%)
Small molecules	68 (100%)	36 (100%)	32 (100%)
Corticosteroids†	68 (100%)	36 (100%)	32 (100%)
History of surgery (n, %)			
Partial colectomy	68 (100%)	36 (100%)	32 (100%)
(Procto)colectomy	68 (100%)	36 (100%)	32 (100%)
Ileocoecal resection	68 (100%)	36 (100%)	32 (100%)
History of smoking (n, %)	68 (100%)	36 (100%)	32 (100%)
Educational status (n, %)	68 (100%)	36 (100%)	32 (100%)
Baseline PROMs			
Fatigue VAS	68 (100%)	36 (100%)	32 (100%)
FACIT	66 (97.06%)	35 (97.22%)	31 (96.88%)
MDSS	64 (94.12%)	35 (97.22%)	29 (90.63%)
SIBDQ	66 (97.06%)	35 (97.22%)	31 (96.88%)
EQ-5D-5L	66 (97.06%)	35 (97.22%)	31 (96.88%)
PSS	64 (94.12%)	35 (97.22%)	29 (90.63%)
B-IPQ	64 (94.12%)	35 (97.22%)	29 (90.63%)
PSQI	64 (94.12%)	35 (97.22%)	29 (90.63%)
iMCQ	63 (92.65%)	35 (97.22%)	28 (87.50%)
SQUASH	58 (85.29%)	31 (97.22%)	27 (84.38%)
Health Monitor	66 (97.06%)	35 (97.22%)	31 (96.88%)
WPAI	64 (94.12%)	35 (97.22%)	29 (90.63%)
3 months - PROMs	*n* = 65	*n* = 33	*n* = 32
Fatigue VAS	57 (87.69%)	31 (93.94%)	26 (81.25%)
FACIT	57 (87.69%)	31 (93.94%)	26 (81.25%)
MDSS	57 (87.69%)	31 (93.94%)	26 (81.25%)
SIBDQ	57 (87.69%)	31 (93.94%)	26 (81.25%)
EQ-5D-5L	57 (87.69%)	31 (93.94%)	26 (81.25%)
PSS	57 (87.69%)	31 (93.94%)	26 (81.25%)
B-IPQ	57 (87.69%)	31 (93.94%)	26 (81.25%)
PSQI	57 (87.69%)	31 (93.94%)	26 (81.25%)
iMCQ	55 (84.62%)	31 (93.94%)	24 (75.00%)
SQUASH	51 (78.46%)	28 (84.85%)	23 (71.88%)
6 months - PROMs	*n* = 63	*n* = 32	*n* = 31
Fatigue VAS	52 (82.54%)	28 (87.50%)	24 (77.42%)
FACIT	52 (82.54%)	28 (87.50%)	24 (77.42%)

MDSS	51 (80.95%)	28 (87.50%)	23 (74.19%)
SIBDQ	52 (82.54%)	28 (87.50%)	24 (77.42%)
EQ-5D-5L	52 (82.54%)	28 (87.50%)	24 (77.42%)
PSS	51 (80.95%)	28 (87.50%)	23 (74.19%)
B-IPQ	51 (80.95%)	28 (87.50%)	23 (74.19%)
PSQI	51 (80.95%)	28 (87.50%)	23 (74.19%)
iMCQ	45 (71.43%)	26 (81.25%)	19 (61.29%)
SQUASH	45 (71.43%)	24 (75.00%)	21 (67.74%)
Health Monitor	52 (82.54%)	28 (87.50%)	24 (77.42%)
WPAI	52 (82.54%)	28 (87.50%)	24 (77.42%)
12 months - PROMs	*n* = 62	*n* = 31	*n* = 31
Fatigue VAS	51 (82.26%)	27 (87.10%)	24 (77.42%)
FACIT	51 (82.26%)	27 (87.10%)	24 (77.42%)
MDSS	51 (82.26%)	28 (90.32%)	23 (74.19%)
SIBDQ	51 (82.26%)	27 (87.10%)	24 (77.42%)
EQ-5D-5L	51 (82.26%)	27 (87.10%)	24 (77.42%)
PSS	51 (82.26%)	28 (90.32%)	2323 (74.19%)
B-IPQ	51 (82.26%)	28 (90.32%)	23 (74.19%)
PSQI	51 (82.26%)	28 (90.32%)	23 (74.19%)
iMCQ	51 (82.26%)	28 (90.32%)	23 (74.19%)
SQUASH	51 (82.26%)	28 (90.32%)	23 (74.19%)
Health Monitor	51 (82.26%)	27 (87.10%)	24 (77.42%)
WPAI	51 (82.26%)	28 (90.32%)	23 (74.19%)

*Data are presented as absolute numbers of available data (n) with corresponding percentages (%); 100% available data indicates no missing values for a given variable. Adjusted MDSS: adjusted Mediterranean Diet Serving Score; BMI: body mass index; B-IPQ: the Brief Illness Perception Questionnaire assesses the cognitive and emotional representations of illness; EQ-5D-5L: a survey assessing quality of life; FACIT-F: The Functional Assessment of Chronic Illness Therapy – Fatigue; GI: gastrointestinal tract; IBD: Inflammatory Bowel Disease; iMCQ: the iMTA Medical Consumption Questionnaire; PSS: PROMs: patient-reported outcome measures; Perceived Stress Scale; PSQI: Pittsburgh Sleep Quality Index; SIBDQ: Short Inflammatory Bowel Disease Questionnaire; SQUASH: Short Questionnaire to Assess Health-enhancing physical activity; WPAI: Work Productivity and Activity Impairment. ‡Includes patients with ulcerative colitis and one patient with IBD-Unclassified. *Upper GI-involvement (Montreal L4) is presented as a modifier, which includes isolated upper GI-involvement and upper GI-involvement in addition to other disease locations. **Perianal disease (Montreal p) is presented as a modifier—indicating solely perianal disease involvement separate from penetrating disease behavior. †Systemic corticosteroids.*

Supplementary Table 3: Baseline clinical characteristics of the study population, before Inverse Probability of Treatment Weighing

	Before the IPTW			
	**Intervention**	**Control**	***P*-value**§	**SMD**
Female sex (n, %)	19 (52.80%)	15 (46.90%)	0.627	0.117
Age (years)	42.50 [32.00–51.00]	37.50 [27.75–46.00]	0.282	-0.323
BMI (kg/m^2)	25.19 [22.39–28.55]	24.73 [21.66–28.57]	0.606	-0.095
IBD disease duration (years)	15.00 [9.00–24.25]	11.50 [3.25–18.00]	0.038	-0.553
IBD type: UC (n, %)	14 (38.90%)	11 (34.40%)	0.700	-0.092
Fecal calprotectin (µg/g)	22.00 [12.00–52.00]	25.50 [13.00–47.50]	0.925	-0.087
C-reactive protein (mg/L)	1.65 [0.80–3.93]	2.10 [1.00–4.45]	0.602	0.260
Ulcerative colitis‡ (n, %)				
Age of onset (n, %)				
<17 years old	4 (28.60%)	3 (27.30%)		
17–40 years old	9 (64.30%)	6 (54.50%)		
>40 years old	1 (7.10%)	2 (18.20%)		
Disease extension (n, %)			0.356	0.514
Pancolitis	4 (28.60%)	5 (45.50%)		
Left-sided colitis	8 (57.10%)	6 (54.50%)		
Proctitis	2 (14.30%)	0 (0.00%)		
Crohn's disease (n, %)				
Age of onset (n, %)				
<17 years old	3 (13.60%)	5 (23.80%)		
17–40 years old	16 (72.70%)	13 (61.90%)		
>40 years old	3 (13.60%)	3 (14.30%)		
Disease location (n, %)			0.722	0.242
Terminal ileum	11 (50.00%)	8 (38.10%)		
Colon	2 (9.10%)	2 (9.50%)		
Ileocolon	9 (40.90%)	11 (52.40%)		
*Upper-GI disease**	4 (18.20%)	4 (19.00%)	0.942	0.022
*Perianal disease***	8 (36.40%)	5 (23.80%)	0.370	-0.269
Disease behavior (n, %)			0.041	-0.809
Inflammatory	10 (45.50%)	17 (81.00%)		
Stricturing	6 (27.30%)	3 (14.30%)		
Penetrating	6 (27.30%)	1 (4.80%)		
Medication (n, %)				
Aminosalicylates	10 (27.80%)	8 (25.00%)	0.796	-0.062
Immunomodulators	10 (27.80%)	11 (34.40%)	0.557	0.141
Biologics	21 (58.30%)	20 (62.50%)	0.726	0.084
Small molecules	0 (0.00%)	1 (3.10%)	0.285	0.258
Corticosteroids†	1 (2.80%)	3 (9.40%)	0.248	0.279
History of surgery (n, %)				
(Procto)colectomy	2 (5.60%)	1 (3.10%)	0.626	-0.117
Ileocoecal resection	8 (22.20%)	4 (12.50%)	0.294	-0.253
History of smoking (n, %)			0.005	0.174
Never	21 (58.30%)	21 (65.60%)		
Current	0 (0.00%)	6 (18.80%)		
Former	15 (41.70%)	5 (15.60%)		
Educational status (n, %)			0.505	0.236
Primary education	1 (2.80%)	0 (0.00%)		
Secondary education	16 (44.40%)	12 (37.50%)		
Higher education	19 (52.80%)	20 (62.50%)		

*Initially, 40 participants were allocated to the intervention group and 32 to the control group; four participants dropped out at or just after baseline. Consequently, 36 participants in the intervention group and 32 in the control group were compared. For an overview of available and missing data per outcome measure refer to Supplementary Table S2. Data are presented as median [interquartile ranges] or as absolute numbers of available data (n) with corresponding percentages (%). BMI: body mass index; GI: gastrointestinal tract; IBD: Inflammatory Bowel Disease; IPTW: Inverse Probability of Treatment Weighing; SMD: Standardized Mean Difference; UC: ulcerative colitis. ‡Includes patients with ulcerative colitis and one patient with IBD-Unclassified. *Upper GI-involvement (Montreal L4) is presented as a modifier, which includes isolated upper GI-involvement and upper GI-involvement in addition to other disease locations. **Perianal disease (Montreal p) is presented as a modifier—indicating solely perianal disease involvement separate from penetrating disease behavior. †Systemic corticosteroids. Two-tailed P-values <0.05 were considered statistically significant; the Benjamini-Hochberg procedure was used to adjust for multiple testing, adopting a 5% false discovery rate (FDR). §P-values were not statistically significant after adjustment for multiple testing.*

Supplementary Table 4: Baseline patient-reported outcome measures for the study population.

	Before the IPTW				After the IPTW			
	**Intervention**	**Control**	***P*-value**	**SMD**	**Intervention**	**Control**	***P*-value**	**SMD**
Fatigue								
Fatigue VAS	6.50 [5.00–7.00]	5.00 [5.00–6.00]	0.017	-0.609	6.00 [4.75–7.00]	6.00 [5.00–7.00]	0.273	0.006
FACIT-F	98.00 [86.00–112.17]	113.00 [96.00–124.00]	0.026	0.583	106.46 [90.09–121.61]	106.00 [88.00–116.86]	0.396	-0.035
Physical Well-Being	21.00 [17.00–24.00]	23.00 [19.00–24.00]	0.172	0.362	21.00 [18.43–24.00]	21.12 [16.44–24.00]	0.918	-0.096
Social Well-Being	21.00 [17.00–22.00]	20.00 [18.00–23.00]	0.466	0.212	21.00 [17.00–23.09]	21.00 [18.00–23.23]	0.475	0.072
Emotional Well-Being	19.00 [16.00–20.00]	20.00 [18.00–22.00]	0.035	0.416	19.00 [16.80–20.00]	19.00 [15.00–21.00]	0.358	-0.101
Functional Well-Being	16.00 [13.00–20.00]	20.00 [17.00–21.00]	0.017	0.624	17.86 [14.03–21.61]	18.74 [17.00–20.00]	0.111	0.141
Fatigue	26.00 [20.00–30.00]	32.00 [22.00–39.00]	0.032	0.561	27.00 [22.00–32.53]	26.51 [16.00–33.91]	0.552	-0.080
Quality of life								
EQ-5D index	0.79 [0.70–0.87]	0.82 [0.77–0.89]	0.288	0.224	0.85 [0.73–0.88]	0.80 [0.70–0.89]	0.980	-0.108
EQ-5D VAS	63.00 [52.00–72.00]	75.00 [66.00–80.00]	**0.001**	0.764	66.30 [60.00–74.95]	70.30 [52.64–80.00]	0.050	0.245
SIBDQ	49.00 [41.00–55.00]	52.00 [44.00–60.00]	0.183	0.302	50.00 [44.00–57.00]	49.81 [43.00–56.00]	1.000	-0.161
Disease perception								
B-IPQ	42.00 [32.00–47.00]	34.00 [26.50–42.00]	0.012	-0.667	40.00 [31.22–46.14]	37.20 [28.07–48.00]	0.313	-0.175
Acceptance	3.33 [2.67–4.33]	4.33 [3.33–5.67]	0.009	0.716	3.84 [2.77–4.96]	3.34 [2.67–4.91]	0.265	-0.001
Perceived control	4.33 [3.67–5.00]	4.67 [4.00–5.67]	0.050	0.440	4.33 [3.81–5.24]	4.33 [3.67–5.23]	0.280	-0.011
Health Monitor segment			0.046	-0.720			0.289	-0.135
Segment 1	1 (2.90%)	7 (22.60%)			1 (3.00%)	5 (13.50%)		
Segment 2	2 (5.70%)	4 (12.90%)			4 (12.10%)	3 (8.10%)		
Segment 3	6 (17.10%)	5 (16.10%)			7 (21.20%)	4 (10.80%)		
Segment 4	26 (74.30%)	15 (48.40%)			21 (63.60%)	25 (67.60%)		
Productivity								
WPAI: Absenteeism*	5 (19.20%)	4 (17.40%)	0.868	-0.047	4 (14.80%)	7 (28.00%)	0.245	0.289
WPAI: Presenteeism	30.00 [7.50–52.50]	20.00 [10.00–30.00]	0.156	-0.491	30.00 [0.00–50.00]	20.00 [10.00–31.99]	0.379	-0.184
WPAI: Activity impairment	50.00 [30.00–70.00]	30.00 [10.00–50.00]	0.017	-0.607	42.38 [30.00–70.00]	40.00 [20.00–60.00]	0.133	-0.204
Lifestyle								
Adjusted MDSS	6.00 [5.00–7.00]	6.00 [5.00–8.00]	0.593	0.209	6.00 [5.00–7.00]	6.00 [5.00–8.00]	0.318	0.272
PSS	16.00 [11.00–20.00]	15.00 [10.50–18.00]	0.685	-0.142	14.47 [10.66–18.00]	17.18 [12.00–19.00]	0.613	0.258
PSQI	6.00 [5.00–11.00]	6.00 [4.00–12.00]	0.946	0.075	5.57 [4.00–8.71]	8.00 [5.00–12.00]	0.251	0.466

*Initially, 40 participants were allocated to the intervention group and 32 to the control group; four participants dropped out at or just after baseline. Consequently, 36 participants in the intervention group and 32 in the control group were compared. For an overview of available and missing data per outcome measure refer to Supplementary Table S2. Data are presented as median [interquartile ranges] or as absolute numbers of available data (n) with corresponding percentages (%). Adjusted MDSS: adjusted Mediterranean Diet Serving Score evaluates dietary habits and adherence to the Mediterranean diet, higher scores indicate better adherence. B-IPQ: the Brief Illness Perception Questionnaire assesses the cognitive and emotional representations of illness; higher scores indicate that a disease is perceived as a health threat. EQ-5D index: index represents health utility ranging from 0.0 (dead) to 1.0 (perfect health). EQ-5D VAS: visual analog scale ranging from 0 (worst health) to 100 (best health). Fatigue VAS: visual analog scale ranging from 0 (no fatigue) to 10 (severe fatigue). FACIT-F: The Functional Assessment of Chronic Illness Therapy – Fatigue, lower scores indicate more severe fatigue. Health Monitor questionnaire evaluates subjective health experience expressed as a perceived sense of control and acceptance; lower scores indicate lower acceptance and a lower perceived sense of control. Segment 1 indicates high perceived control and high acceptance; segment 2 indicates low perceived control and a high acceptance score; segment 3 indicates a high score of perceived control and a low acceptance score; segment 4 indicates low perceived control and a low acceptance score. IPTW: Inverse Probability of Treatment Weighing. PSS: Perceived Stress Scale evaluates perceived stress with scores ranging from 0 (no perceived stress) to 40 (high perceived stress). PSQI: Pittsburgh Sleep Quality Index assesses sleep quality, with higher scores indicating worse sleep quality. SIBDQ: Short Inflammatory Bowel Disease Questionnaire assesses health-related quality of life; higher scores indicate better health-related quality of life. SMD: Standardized Mean Difference. WPAI: Work Productivity and Activity Impairment assesses work and activity impairment with higher scores indicating higher productivity loss; Presenteeism indicates impairment at work, whereas Activity impairment represents impairment during daily activities outside work, with higher scores indicating worse impairment. *Indicates the number of patients who were unable to attend work due to complaints related to Inflammatory Bowel Disease. Two-tailed P-values <0.05 were considered statistically significant; the Benjamini-Hochberg procedure was used to adjust for multiple testing, adopting a 5% false discovery rate (FDR). P-values in **bold** indicate statistical significance after adjustment for multiple testing.*

Supplementary Table 5: Changes in patient-reported outcome measures after three months of intervention, stratified by treatment type.

	**Before the Inverse Probability of Treatment Weighing**									
	**Intervention**				**Control**					
	**Baseline**	**After three months**	***P*-value**	**SMD**	**Baseline**	**After three months**	***P*-value**	**SMD**	***P*-value for Δ**	**SMD for Δ**
Fatigue VAS	6.50 [5.00–7.00]	6.00 [4.00–7.00]	0.041	0.370	5.00 [5.00–6.00]	6.00 [4.00–7.25]	0.728	-0.043	0.143	-0.440
FACIT-F	98.00 [86.00–112.17]	107.00 [96.00–129.00]	**<0.001**	-0.660	113.00 [96.00–124.00]	115.50 [98.00–128.00]	0.943	-0.001	**0.006**	0.772
Physical Well-Being	21.00 [17.00–24.00]	23.00 [19.00–25.00]	0.021	-0.495	23.00 [19.00–24.00]	23.00 [20.50–25.00]	0.326	-0.080	0.223	0.470
Social Well-Being	21.00 [17.00–22.00]	21.00 [19.00–23.33]	0.035	-0.381	20.00 [18.00–23.00]	21.00 [18.00–22.17]	0.766	-0.002	0.097	0.411
Emotional Well-Being	19.00 [16.00–20.00]	19.00 [16.00–21.00]	0.135	-0.234	20.00 [18.00–22.00]	20.50 [17.75–22.00]	0.409	-0.143	0.379	0.149
Functional Well-Being	16.00 [13.00–20.00]	18.00 [14.00–20.00]	0.405	-0.192	20.00 [17.00–21.00]	20.00 [15.75–21.00]	0.057	0.402	0.057	0.555
Fatigue	26.00 [20.00–30.00]	31.00 [24.00–39.00]	**0.001**	-0.670	32.00 [22.00–39.00]	33.50 [21.50–39.75]	0.828	-0.092	0.022	0.672
EQ-5D index	0.79 [0.70–0.87]	0.81 [0.72–0.89]	0.542	-0.114	0.82 [0.77–0.89]	0.82 [0.75–0.88]	0.616	0.019	0.791	0.134
EQ-5D VAS	63.00 [52.00–72.00]	70.00 [50.00–80.00]	0.070	-0.284	75.00 [66.00–80.00]	70.50 [59.75–78.50]	0.085	0.344	0.025	0.623
SIBDQ	49.00 [41.00–55.00]	51.00 [46.00–57.00]	0.044	-0.374	52.00 [44.00–60.00]	52.00 [47.00–56.50]	0.833	-0.031	0.196	0.353
B-IPQ	42.00 [32.00–47.00]	36.00 [29.00–44.00]	**<0.001**	0.737	34.00 [26.50–42.00]	33.00 [25.00–40.50]	0.626	0.095	0.013	-0.706
Adjusted MDSS	6.00 [5.00–7.00]	10.00 [8.00–10.00]	**<0.001**	-1.389	6.00 [5.00–8.00]	6.00 [5.00–7.25]	0.947	<0.001	**<0.001**	1.636
PSS	16.00 [11.00–20.00]	13.00 [9.00–18.00]	0.321	0.157	15.00 [10.50–18.00]	12.50 [8.50–18.50]	0.787	0.106	0.619	-0.079
PSQI	6.00 [5.00–11.00]	6.00 [4.00–10.00]	0.246	0.220	6.00 [4.00–12.00]	5.50 [4.00–10.50]	0.735	0.051	0.615	-0.169
	**After the Inverse Probability of Treatment Weighing**									
	**Intervention**				**Control**					
	**Baseline**	**After three months**	***P*-value**	**SMD**	**Baseline**	**After three months**	***P*-value**	**SMD**	***P*-value for Δ**	**SMD for Δ**
Fatigue VAS	6.00 [4.75–7.00]	6.00 [3.11–7.00]	0.034	0.313	6.00 [5.00–7.00]	7.00 [5.00–8.00]	0.753	-0.025	0.097	-0.357
FACIT-F	106.46 [90.09–121.61]	110.02 [96.75–132.92]	**0.001**	-0.546	106.00 [88.00–116.86]	113.00 [86.00–121.29]	0.330	-0.138	0.019	0.551
Physical Well-Being	21.00 [18.43–24.00]	23.55 [19.67–25.00]	0.036	-0.384	21.12 [16.44–24.00]	22.67 [18.00–25.00]	0.132	-0.140	0.487	0.288
Social Well-Being	21.00 [17.00–23.09]	21.94 [19.80–24.00]	0.033	-0.293	21.00 [18.00–23.23]	21.00 [18.00–23.12]	0.458	-0.035	0.137	0.291
Emotional Well-Being	19.00 [16.80–20.00]	19.00 [17.00–22.00]	0.151	-0.186	19.00 [15.00–21.00]	19.00 [16.00–21.00]	0.076	-0.353	0.951	-0.129
Functional Well-Being	17.86 [14.03–21.61]	18.09 [15.00–20.38]	0.570	-0.050	18.74 [17.00–20.00]	18.00 [12.54–20.00]	0.010	0.497	0.029	0.531
Fatigue	27.00 [22.00–32.53]	32.49 [25.00–40.00]	**<0.001**	-0.624	26.51 [16.00–33.91]	29.00 [16.89–36.97]	0.226	-0.240	0.046	0.504
EQ-5D index	0.85 [0.73–0.88]	0.84 [0.73–0.89]	0.882	-0.012	0.80 [0.70–0.89]	0.81 [0.72–0.87]	0.130	-0.136	0.552	-0.117
EQ-5D VAS	66.30 [60.00–74.95]	70.00 [57.20–80.00]	0.084	-0.223	70.30 [52.64–80.00]	70.00 [50.00–76.25]	0.229	0.154	0.082	0.376
SIBDQ	50.00 [44.00–57.00]	51.92 [46.57–59.87]	**0.017**	-0.471	49.81 [43.00–56.00]	52.00 [47.00–55.19]	0.180	-0.317	0.467	0.119
B-IPQ	40.00 [31.22–46.14]	34.08 [26.00–39.81]	**<0.001**	0.876	37.20 [28.07–48.00]	35.04 [27.58–48.53]	0.641	0.123	**0.003**	-0.779
Adjusted MDSS	6.00 [5.00–7.00]	10.00 [7.62–10.00]	**<0.001**	-1.327	6.00 [5.00–8.00]	6.00 [5.00–7.00]	0.397	0.154	**<0.001**	1.706
PSS	14.47 [10.66–18.00]	13.00 [8.41–17.00]	0.223	0.221	17.18 [12.00–19.00]	14.00 [11.00–20.04]	0.258	0.219	0.995	-0.022
PSQI	5.57 [4.00–8.71]	5.00 [4.00–9.00]	0.167	0.249	8.00 [5.00–12.00]	7.00 [4.21–14.00]	0.680	-0.046	0.272	-0.297

Initially, 40 participants were allocated to the intervention group and 32 to the control group; four participants dropped out at or just after baseline. Consequently, 36 participants in the intervention group and 32 in the control group were compared. Six months after the baseline, there were 32 participants in the intervention and 31 in the control group. For an overview of available and missing data per outcome measure refer to Supplementary Table S2. Data are presented as median [interquartile ranges] or as absolute numbers of available data (n) with corresponding percentages (%). Adjusted MDSS: adjusted Mediterranean Diet Serving Score evaluates dietary habits and adherence to the Mediterranean diet,

higher scores indicate better adherence. B-IPQ: the Brief Illness Perception Questionnaire assesses the cognitive and emotional representations of illness; higher scores indicate that a disease is perceived as a health threat. EQ-5D index: index represents health utility ranging from 0.0 (dead) to 1.0 (perfect health). EQ-5D VAS: visual analog scale ranging from 0 (worst health) to 100 (best health). Fatigue VAS: visual analog scale ranging from 0 (no fatigue) to 10 (severe fatigue). FACIT-F: The Functional Assessment of Chronic Illness Therapy – Fatigue, lower scores indicate more severe fatigue. PSS: Perceived Stress Scale evaluates perceived stress with scores ranging from 0 (no perceived stress) to 40 (high perceived stress). PSQI: Pittsburgh Sleep Quality Index assesses sleep quality, with higher scores indicating worse sleep quality. SIBDQ: Short Inflammatory Bowel Disease Questionnaire assesses health-related quality of life; higher scores indicate better health-related quality of life. SMD: Standardized Mean Difference. Two-tailed P-values <0.05 were considered statistically significant; the Benjamini-Hochberg procedure was used to adjust for multiple testing, adopting a 5% false discovery rate (FDR). P-values in ***bold*** *indicate statistical significance after adjustment for multiple testing. The P-value for Δ indicates the statistical difference in changes between the treatment groups.*

Supplementary Table 6: Changes and differences between treatment groups regarding patient-reported outcomes after six months of intervention.

	**Before the Inverse Probability of Treatment Weighing**									
	**Intervention**				**Control**					
	**Baseline**	**After six months**	***P*-value**	**SMD**	**Baseline**	**After six months**	***P*-value**	**SMD**	***P*-value for Δ**	**SMD for Δ**
Fatigue VAS	6.50 [5.00–7.00]	6.00 [4.00–7.00]	**0.017**	0.505	5.00 [5.00–6.00]	5.00 [3.25–7.00]	0.216	0.239	0.387	-0.305
EQ-5D index	0.79 [0.70–0.87]	0.81 [0.68–0.91]	0.414	-0.084	0.82 [0.77–0.89]	0.82 [0.72–0.89]	0.958	0.040	0.263	0.128
SIBDQ	49.00 [41.00–55.00]	50.50 [45.00–60.50]	0.109	-0.345	52.00 [44.00–60.00]	52.00 [44.25–58.75]	0.439	0.118	0.136	0.469
WPAI										
Presenteeism	30.00 [7.50–52.50]	10.00 [10.00–30.00]	0.111	0.391	20.00 [10.00–30.00]	10.00 [0.00–30.00]	0.839	-0.138	0.117	-0.556
Activity impairment	50.00 [30.00–70.00]	30.00 [20.00–60.00]	0.072	0.390	30.00 [10.00–50.00]	25.00 [10.00–50.00]	0.742	0.026	0.217	-0.441
PSS	16.00 [11.00–20.00]	17.50 [11.25–20.00]	0.893	<0.001	15.00 [10.50–18.00]	14.00 [8.00–20.00]	0.695	-0.070	0.776	-0.070
PSQI	6.00 [5.00–11.00]	5.50 [3.00–11.00]	0.117	0.149	6.00 [4.00–12.00]	5.00 [4.00–9.00]	0.060	0.440	0.696	0.203
	**After the Inverse Probability of Treatment Weighing**									
	**Intervention**				**Control**					
	**Baseline**	**After six months**	***P*-value**	**SMD**	**Baseline**	**After six months**	***P*-value**	**SMD**	***P*-value for Δ**	**SMD for Δ**
Fatigue VAS	6.00 [4.75–7.00]	6.00 [4.00–7.00]	**0.010**	0.501	6.00 [5.00–7.00]	7.00 [4.00–9.00]	0.575	0.031	0.071	-0.494
EQ-5D index	0.85 [0.73–0.88]	0.86 [0.70–1.00]	0.414	-0.145	0.80 [0.70–0.89]	0.76 [0.67–0.87]	0.902	0.037	0.283	0.199
SIBDQ	50.00 [44.00–57.00]	51.76 [45.62–61.00]	0.094	-0.403	49.81 [43.00–56.00]	48.12 [42.00–56.00]	1.000	-0.50	0.286	0.347
WPAI										
Presenteeism	30.00 [0.00–50.00]	10.00 [10.00–30.00]	0.094	0.383	20.00 [10.00–31.99]	20.00 [1.44–40.00]	0.338	-0.328	0.038	-0.713
Activity impairment	42.38 [30.00–70.00]	30.00 [10.00–57.49]	0.042	0.410	40.00 [20.00–60.00]	48.64 [15.52–70.00]	0.731	-0.066	0.069	-0.552
PSS	14.47 [10.66–18.00]	17.00 [10.76–20.00]	0.770	-0.018	17.18 [12.00–19.00]	17.00 [8.77–22.00]	0.918	-0.105	0.933	-0.084
PSQI	5.57 [4.00–8.71]	5.50 [3.00–11.00]	0.117	0.170	8.00 [5.00–12.00]	8.00 [4.00–10.98]	0.004	0.513	0.268	0.251

Initially, 40 participants were allocated to the intervention group and 32 to the control group; four participants dropped out at or just after baseline. Consequently, 36 participants in the intervention group and 32 in the control group were compared. Six months after the baseline, there were 32 participants in the intervention and 31 in the control group. For an overview of available and missing data per outcome measure refer to Supplementary Table S2. Data are presented as median [interquartile ranges] or as absolute numbers of available data (n) with corresponding percentages (%). EQ-5D index: index represents health utility ranging from 0.0 (dead) to 1.0 (perfect health). Fatigue VAS: visual analog scale ranging from 0 (no fatigue) to 10 (severe fatigue). PSS: Perceived Stress Scale evaluates perceived stress with scores ranging from 0 (no perceived stress) to 40 (high perceived stress). PSQI: Pittsburgh Sleep Quality Index assesses sleep quality, with higher scores indicating worse sleep quality. SIBDQ: Short Inflammatory Bowel Disease Questionnaire assesses health-related quality of life; higher scores indicate better health-related quality of life. SMD: Standardized Mean Difference. WPAI: Work Productivity and Activity Impairment assesses work and activity impairment with higher scores indicating higher productivity loss; Presenteeism indicates impairment at work, whereas Activity impairment represents impairment during daily activities outside work, with higher scores indicating worse impairment. Two-tailed P-values <0.05 were considered statistically significant; the Benjamini-Hochberg procedure was used to adjust for multiple testing, adopting a 5% false discovery rate (FDR). P-values in ***bold*** *indicate statistical significance after adjustment for multiple testing. The P-value for Δ indicates the statistical difference in changes between the treatment groups.*

Supplementary Table 7: Changes and differences in patient-reported outcome measures after 12 months of intervention, stratified by treatment type.

	**Before the Inverse Probability of Treatment Weighing**									
	**Intervention**				**Control**					
	**Baseline**	**After 12 months**	***P*-value**	**SMD**	**Baseline**	**After 12 months**	***P*-value**	**SMD**	***P*-value for Δ**	**SMD for Δ**
Fatigue VAS	6.50 [5.00–7.00]	6.00 [4.00–7.00]	0.038	0.402	5.00 [5.00–6.00]	6.00 [4.00–7.00]	0.606	0.121	0.197	-0.350
EQ-5D index	0.79 [0.70–0.87]	0.84 [0.78–0.89]	0.767	0.006	0.82 [0.77–0.89]	0.82 [0.73–0.89]	0.816	-0.186	0.985	-0.189
SIBDQ	49.00 [41.00–55.00]	50.00 [45.00–57.00]	0.220	-0.228	52.00 [44.00–60.00]	53.50 [45.25–59.00]	0.807	0.013	0.311	0.263
WPAI										
Presenteeism	30.00 [7.50–52.50]	10.00 [0.00–40.00]	0.130	0.339	20.00 [10.00–30.00]	10.00 [0.00–45.00]	0.877	-0.116	0.255	-0.463
Activity impairment	50.00 [30.00–70.00]	35.00 [10.00–50.00]	**0.003**	0.681	30.00 [10.00–50.00]	30.00 [10.00–60.00]	0.718	0.021	0.043	-0.665
PSS	16.00 [11.00–20.00]	14.50 [9.00–20.50]	0.718	0.048	15.00 [10.50–18.00]	13.00 [8.00–18.00]	0.574	0.184	0.953	0.073
PSQI	6.00 [5.00–11.00]	6.00 [3.00–10.00]	0.137	0.341	6.00 [4.00–12.00]	5.00 [5.00–11.00]	0.361	0.194	0.128	-0.212
	**After the Inverse Probability of Treatment Weighing**									
	**Intervention**				**Control**					
	**Baseline**	**After 12 months**	***P*-value**	**SMD**	**Baseline**	**After 12 months**	***P*-value**	**SMD**	***P*-value for Δ**	**SMD for Δ**
Fatigue VAS	6.00 [4.75–7.00]	6.00 [4.25–7.00]	0.060	0.213	6.00 [5.00–7.00]	6.00 [4.00–7.00]	0.774	0.112	0.149	-0.152
EQ-5D index	0.85 [0.73–0.88]	0.85 [0.80–0.89]	0.970	-0.002	0.80 [0.70–0.89]	0.78 [0.65–0.88]	0.432	-0.229	0.552	-0.230
SIBDQ	50.00 [44.00–57.00]	50.00 [45.02–57.00]	0.289	-0.055	49.81 [43.00–56.00]	49.47 [43.00–55.00]	0.971	-0.068	0.365	0.009
WPAI										
Presenteeism	30.00 [0.00–50.00]	10.00 [0.00–48.04]	0.251	0.133	20.00 [10.00–31.99]	26.20 [8.01–50.00]	0.569	-0.159	0.282	-0.290
Activity impairment	42.38 [30.00–70.00]	38.06 [10.00–50.00]	**0.003**	0.513	40.00 [20.00–60.00]	30.00 [10.00–80.00]	0.881	0.012	0.019	-0.491
PSS	14.47 [10.66–18.00]	12.00 [5.00–18.87]	0.657	0.046	17.18 [12.00–19.00]	15.00 [11.39–18.00]	0.167	0.306	0.739	0.187
PSQI	5.57 [4.00–8.71]	5.00 [3.00–9.54]	0.086	0.390	8.00 [5.00–12.00]	6.46 [5.00–12.00]	0.919	0.008	0.021	-0.406

Initially, 40 participants were allocated to the intervention group and 32 to the control group; four participants dropped out at or just after baseline. Consequently, 36 participants in the intervention group and 32 in the control group were compared. Six months after the baseline, there were 32 participants in the intervention and 31 in the control group. For an overview of available and missing data per outcome measure refer to Supplementary Table S2. Data are presented as median [interquartile ranges] or as absolute numbers of available data (n) with corresponding percentages (%). EQ-5D index: index represents health utility ranging from 0.0 (dead) to 1.0 (perfect health). Fatigue VAS: visual analog scale ranging from 0 (no fatigue) to 10 (severe fatigue). PSS: Perceived Stress Scale evaluates perceived stress with scores ranging from 0 (no perceived stress) to 40 (high perceived stress). PSQI: Pittsburgh Sleep Quality Index assesses sleep quality, with higher scores indicating worse sleep quality. SIBDQ: Short Inflammatory Bowel Disease Questionnaire assesses health-related quality of life; higher scores indicate better health-related quality of life. SMD: Standardized Mean Difference. WPAI: Work Productivity and Activity Impairment assesses work and activity impairment with higher scores indicating higher productivity loss; Presenteeism indicates impairment at work, whereas Activity impairment represents impairment during daily activities outside work, with higher scores indicating worse impairment. Two-tailed P-values <0.05 were considered statistically significant; the Benjamini-Hochberg procedure was used to adjust for multiple testing, adopting a 5% false discovery rate (FDR). P-values in ***bold*** *indicate statistical significance after adjustment for multiple testing. The P-value for Δ indicates the statistical difference in changes between the treatment groups.*

Supplementary Table 8: Overview of adverse events.

	**Adverse events**	
	**Intervention**	**Control**
Infections (n, %)		
Respiratory infections	2 (5.56%)	4 (12.50%)
Gastrointestinal infections	4 (11.11%)	2 (6.25%)
Dental alveolitis	-	1 (3.13%)
Herpes zoster reactivation	1 (2.78%)	-
Unspecified	1 (2.78%)	1 (3.13%)
Neuromusculoskeletal complaints (n, %)		
Arthralgia	2 (5.56%)	2 (6.25%)
Muscle pain	2 (5.56%)	1 (3.13%)
Muscle cramps	1 (2.78%)	-
Concussion	1 (2.78%)	-
Ruptured meniscus	1 (2.78%)	-
Hypesthesia	1 (2.78%)	-
IBD exacerbation (n, %)		
Isolated increase in fecal calprotectin	1 (2.78%)	1 (3.13%)
*Mild exacerbation**	4 (11.11%)	2 (6.25%)
*Moderate-to-severe exacerbation***	5 (13.89%)	5 (15.63%)
Gastrointestinal complaints not related to IBD activity (n, %)		
Nausea	4 (11.11%)	2 (6.25%)
Diarrhea	3 (8.33%)	3 (9.38%)
Flatulence	2 (5.56%)	4 (12.50%)
Abdominal pain	8 (22.22%)	8 (25.00%)
Anal fissure	1 (2.78%)	-
Achalasia	1 (2.78%)	-
Dyspepsia	1 (2.78%)	1 (3.13%)
Constipation	-	1 (3.13%)
Hematochezia	-	1 (3.13%)
Loss of appetite	-	1 (3.13%)
Dermatological complaints (n, %)		
Pruritus	2 (5.56%)	1 (3.13%)
Facial erythema	1 (2.78%)	-
Other	1 (2.78%)	-
Hematological abnormalities		
Leukopenia	1 (2.78%)	-
Lymphoma	1 (2.78%)	-
Normocytic anemia	1 (2.78%)	-
Hospitalization (n, %)	6 (16.67%)	2 (6.25%)
Other complaints (n, %)		
Anxiety	1 (2.78%)	-
Depression	1 (2.78%)	-
Fever (non-infectious)	1 (2.78%)	-
Night sweats	1 (2.78%)	-
Dizziness	1 (2.78%)	-
Hidradenitis suppurutiva	1 (2.78%)	-
Vitreous detachment	1 (2.78%)	-
Urinary hesitancy	-	1 (3.13%)
**TOTAL COUNT OF EVENTS**	66	44

**Includes clinical, biochemical, endoscopic, or radiologic disease activity that was treated locally (e.g., corticosteroid enemas) or optimizing trough levels of the systemic medication (e.g., increasing the frequency or the dose of the medication, or switching from intravenous to subcutaneous injections). **Includes clinical, biochemical, endoscopic, or radiologic disease activity that necessitated changing systemic therapy (e.g., change from ustekinumab to vedolizumab).*

Supplementary Table 9: Correlations between changes in fatigue and other patient-reported outcomes over 12-month period.

	ΔFACIT-F	
	**Spearman's rho**	***P*-value**
ΔFatigue VAS	-0.723	***P* < 0.001**
ΔSIBDQ	0.675	***P* < 0.001**
ΔEQ-5D index	0.480	***P* < 0.001**
ΔEQ-5D VAS	0.553	***P* < 0.001**
ΔBIPQ	-0.433	***P* = 0.001**
ΔHealth monitor: acceptance	0.494	***P* < 0.001**
ΔHealth monitor: control	0.275	***P* = 0.043**
ΔPSS	-0.355	***P* = 0.009**
ΔWPAI: activity impairment	-0.343	***P* = 0.012**
ΔWPAI: Presenteeism	-0.595	***P* < 0.001**
ΔAdjusted MDSS	0.413	***P* = 0.002**
ΔPSQI	-0.184	*P* = 0.186

Initially, 40 participants were allocated to the intervention group and 32 to the control group; four participants dropped out at or just after baseline. Consequently, 36 participants in the intervention group and 32 in the control group were compared. For an overview of available and missing data per outcome measure refer to Supplementary Table S2. Data are presented after Inverse Probability of Treatment Weighing; delta values were calculated "Score at 12 months – Score at baseline". Adjusted MDSS: adjusted Mediterranean Diet Serving Score evaluates dietary habits and adherence to the Mediterranean diet, higher scores indicate better adherence. B-IPQ: the Brief Illness Perception Questionnaire assesses the cognitive and emotional representations of illness; higher scores indicate that a disease is perceived as a health threat. EQ-5D index: index represents health utility ranging from 0.0 (dead) to 1.0 (perfect health). EQ-5D VAS: visual analog scale ranging from 0 (worst health) to 100 (best health). Fatigue VAS: visual analog scale ranging from 0 (no fatigue) to 10 (severe fatigue). FACIT-F: The Functional Assessment of Chronic Illness Therapy – Fatigue, lower scores indicate more severe fatigue. Health Monitor questionnaire evaluates subjective health experience expressed as a perceived sense of control and acceptance; lower scores indicate lower acceptance and a lower perceived sense of control. PSS: Perceived Stress Scale evaluates perceived stress with scores ranging from 0 (no perceived stress) to 40 (high perceived stress). PSQI: Pittsburgh Sleep Quality Index assesses sleep quality, with higher scores indicating worse sleep quality. SIBDQ: Short Inflammatory Bowel Disease Questionnaire assesses health-related quality of life; higher scores indicate better health-related quality of life. WPAI: Work Productivity and Activity Impairment assesses work and activity impairment with higher scores indicating higher productivity loss; Presenteeism indicates impairment at work, whereas Activity impairment represents impairment during daily activities outside work, with higher scores indicating worse impairment. Two-tailed P-values <0.05 were considered statistically significant; the Benjamini-Hochberg procedure was used to adjust for multiple testing, adopting a 5% false discovery rate (FDR). P-values in ***bold*** *indicate statistical significance after adjustment for multiple testing.*

Supplementary Table 10: Explanatory factors and predictors of sleep, perceived stress, and disease perception in patients with inflammatory bowel disease.

	**Sleep quality: PSQI**				
	**β**	**SE β**	***t*-value**	***P*-value**	**95% CI**
Intercept	12.760	3.828	3.334	0.001	[5.099–20.422]
Treatment *(reference: control group)*	-2.321	1.153	-2.013	0.047	[-4.613–(-0.028)]
Sex *(reference: male)*	1.489	0.924	1.612	0.113	[-0.362–3.341]
Age	0.053	0.039	1.344	0.185	[-0.026– 0.132]
Bodyweight	-0.077	0.036	-2.159	0.035	[-0.149–(-0.006)]
IBD type (*reference: UC*)	-0.706	0.929	-0.760	0.451	[-2.568–1.157]
Never smoked *(reference: current smoker)*	2.194	1.695	1.294	0.201	[-1.204–5.591]
Former smoker *(reference: current smoker)*	3.211	1.895	1.694	0.096	[-0.587–7.008]
Primary education *(reference: higher education)*	4.100	3.622	1.132	0.263	[-3.169–11.369]
Secondary education *(reference: higher education)*	0.902	0.898	1.004	0.320	[-0.898–2.702]
Health Monitor: acceptance	-1.033	0.386	-2.675	0.010	[-1.808–(-0.259)]
Health Monitor: control	0.296	0.427	0.693	0.491	[-0.561–1.154]
Time	-0.198	0.176	-1.125	0.262	[-0.546–0.150]
Time*Intervention group *(reference: control group)*	-0.090	0.236	-0.380	0.704	[-0.557–0.377]
	**Perceived stress: PSS**				
	**β**	**SE β**	***t*-value**	***P*-value**	**95% CI**
Intercept	39.103	5.974	6.545	<0.001	[27.152–51.055]
Treatment *(reference: control group)*	-1.843	1.951	-0.944	0.347	[-5.707–2.022]
Sex *(reference: male)*	0.650	1.422	0.457	0.649	[-2.201–3.501]
Age	-0.077	0.061	-1.258	0.214	[-0.198–0.045]
Bodyweight	-0.150	0.055	-2.727	0.009	[-0.260–(-0.040)]
IBD type (reference: UC)	-0.091	1.425	-0.064	0.949	[-2.950–2.768]
Never smoked *(reference: current smoker)*	-0.458	2.609	-0.176	0.861	[-5.689–4.773]
Former smoker *(reference: current smoker)*	0.977	2.915	0.335	0.739	[-4.868–6.822]
Primary education *(reference: higher education)*	-6.697	5.506	-1.216	0.230	[-17.761–4.366]
Secondary education *(reference: higher education)*	2.794	1.383	2.020	0.048	[0.021–5.567]
Health Monitor: acceptance	-2.617	0.595	-4.400	<0.001	[-3.810–(-1.425)]
Health Monitor: control	0.241	0.652	0.370	0.713	[-1.068–1.550]
Time	-0.199	0.377	-0.529	0.597	[-0.943–0.544]
Time*Intervention group *(reference: control group)*	0.126	0.506	0.249	0.803	[-0.873–1.125]
	**Disease perception: B-IPQ**				
	**β**	**SE β**	***t*-value**	***P*-value**	**95% CI**
Intercept	71.340	9.195	7.758	<0.001	[52.942–89.738]
Treatment *(reference: control group)*	4.463	2.875	1.552	0.124	[-1.241–10.166]
Sex *(reference: male)*	2.185	2.205	0.991	0.326	[-2.234–6.604]
Age	-0.164	0.094	-1.744	0.087	[-0.353–0.024]
Bodyweight	-0.052	0.085	-0.606	0.547	[-0.223–0.119]
IBD type (reference: UC)	-4.659	2.214	-2.105	0.040	[-9.098–(-0.220)]
Never smoked *(reference: current smoker)*	-4.392	4.046	-1.086	0.282	[-12.501–3.718]
Former smoker *(reference: current smoker)*	-0.939	4.521	-0.208	0.836	[-10.003–8.124]
Primary education *(reference: higher education)*	3.029	8.595	0.352	0.726	[-14.228–20.287]
Secondary education *(reference: higher education)*	1.715	2.144	0.800	0.427	[-2.582–6.011]
Health Monitor: acceptance	-2.230	0.922	-2.418	0.019	[-4.078–(-0.381)]
Health Monitor: control	-2.687	1.016	-2.645	0.011	[-4.725–(-0.648)]
Time	0.154	0.499	0.308	0.759	[-0.832–1.139]
Time*Intervention group *(reference: control group)*	-2.441	0.670	-3.644	<0.001	[-3.765–(-1.118)]

IBD: Inflammatory Bowel Disease. Perceived stress was measured by the Perceived Stress Scale (PSS), with scores ranging from 0 (no perceived stress) to 40 (high perceived stress). Sleep was measured by the Pittsburgh Sleep Quality Index (PSQI), higher scores indicate worse sleep quality. Disease perception was measured by the Brief Illness Perception Questionnaire (B-IPQ), which assesses the cognitive and emotional representations of illness; higher scores indicate that a disease is perceived as a health threat.

Supplementary Table 11: Description of the multimodal lifestyle intervention.

Lifestyle interventions developed by Voeding Leeft®

The organization Voeding Leeft [www.voedingleeft.nl] has developed various group lifestyle programs for chronic conditions, including type 2 diabetes, Multiple Sclerosis (MS), IBD, and rheumatoid arthritis. Voeding Leeft's *Reverse Diabetes2 Now* program is the first program developed by Voeding Leeft in 2014. In 2020, Pot et al.[1] published long term follow-up (i.e., up to 2 years) results of this program; consequently, the program was assessed by the Dutch National Institute for Public Health and the Environment and found to be effective. In 2021, the EU recognized the *Reverse Diabetes2 Now* program as a best practice for type 2 diabetes. In addition, the program was assessed by the Dutch National Health Care Institute in 2022, which determined that the program met the requirements to be reimbursed under the Dutch basic health insurance for eligible participants. *Reverse Diabetes2 Now* is the first specialized combined lifestyle intervention in the Netherlands. Other Voeding Leeft lifestyle programs are based on the *Reverse Diabetes2 Now* program but are adjusted to fit the needs of different patient populations.

Voeding Leeft’s lifestyle programs are based on scientific-evidence and supported by medical specialists in the relevant field. Voeding Leeft investigates the effectiveness of its lifestyle programs in cooperation with research institutions. All programs aim to support durable lifestyle change around nutrition, exercise, sleep, and relaxation. Participants are encouraged to use the information and experiences provided to increase self-management of their health. The behavior change component of the programs is based on the I-change model of behavior change[2]. This model integrates Azjen’s Theory of Planned Behavior, Bandura’s Social Cognitive Theory, Prochaska’s Transtheoretical Model, the Health Belief Model, and goal setting theories[2].

Voeding Leeft’s programs also integrate experiential learning, an educational approach centered around active engagement and hands-on experiences. The participants learn by actively participating in experiences, reflecting on those experiences, and deriving meaning from them. Thereby increasing the patient’s self-management of their health (Figure 1).

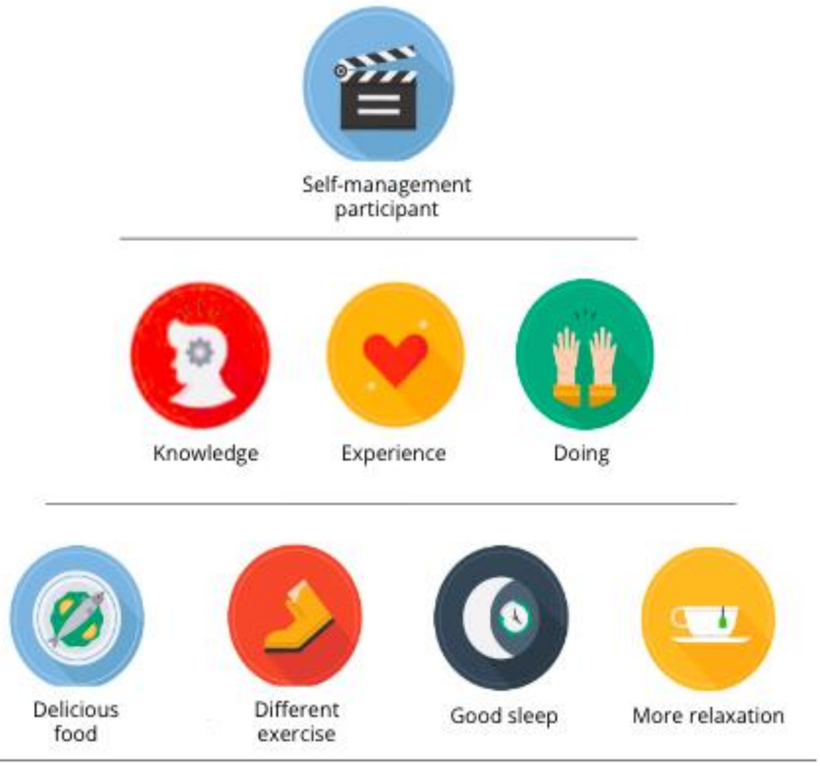

***Figure 1:** the program structure.*

Intervention timeline

In the first six months, the intensive phase, participants follow five online program days (Figure 2). During the

start day, the program pillars and nutritional principles of the program are explained in relation to Crohn's disease and Ulcerative Colitis. The participant support team consists of a nutritionist, lifestyle coach and a moderator. There is ample opportunity for interaction and questions. During the start day, there are plenty of breaks with "*exercise snacks*" and relaxation exercises. Nutritional advice forms the core component for the start day and the first three months. Participants receive information on why they should adopt a varied diet based on unprocessed foods and how this can decrease their IBD symptoms. The nutrition guidelines include a lot of plant-based foods, without excluding food categories such as dairy or grains. In addition, it is recommended to eat three times a day. This allows the body and organs to rest between meals and gives the body the opportunity to recover physiologically. The focus of the diet is on stabilizing the blood sugar level, reducing chronic inflammation and improving gut health (including the composition of the microbiota). Participants receive a four-week meal plan increasing from one to three meals a day, which they are advised to follow closely. The amount of fiber in the diet is gradually increased in order to avoid gastrointestinal complaints. After several weeks, participants are invited to join a live Q&A session with the dietician to discuss successes and challenges with respect to the integration of the diet.

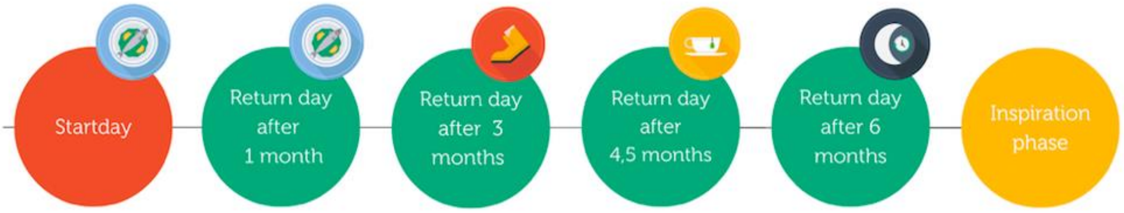

Figure 2: *timeline.*

After the first three months, exercise, stress management, relaxation and sleep are discussed. The relationship between each pillar and IBD complaints is explained. For each of the pillar's participants receive practical exercises to carry out at home. The exercise pillar focuses on what participants are capable of, provides information on how to avoid a sedentary lifestyle and how to exercise in a way that participants enjoy themselves (with an increased heart rate). Additionally, it encourages participants to vary their choice of physical activity. Additional attention is paid for advice regarding coping and adjusting to different limiting factors, e.g., dealing with fatigue, abdominal and/or joint pain. The relaxation pillar helps participants learn to recognize stress signals in the body and acknowledge what stresses them. They are inspired to find a personally effective way to relax daily and ideally multiple times a day. The sleep pillar teaches participants about good sleep quality and the importance of a sleep routine and sleep hygiene.

Alongside the focus on these pillars, coaching also plays a vital role. During coaching sessions, which are spread over the program days, participants talk about their experiences, challenges and disease related themes including acceptance and insecurity in order to integrate behavior change into their daily lives. Participants are supported in formulating their personal goals, facing mental challenges, and breaking habits.

The first 6 months are followed by the 1.5-year maintenance phase. Participation is voluntary. The main goal of this phase is to make the behavior change sustainable, assess difficulties and maintain and/or increase motivation. Participants refresh the knowledge they have received during the intensive phase (first six months); additional coaching sessions are available.

A key principle of the group intervention is that participants stimulate, support, and learn from each other's experiences. In other words, the group process plays a crucial role. This happens both during the program days and in the online community. During the 2-year program, all participants can access an online community 24/7. In this community, they can find all relevant information, stay in touch with other participants and the support team, and track their own progress. Responses from participants and their interactions are actively monitored and moderated in this community.

Intervention materials

After the first program day, participants receive information to get started via the online community. This includes the presentations given during the online start day and a menu with recipes for the first four weeks. This four-week menu is developed by the nutritionist based on the previously mentioned principles. The menu

is introduced using a stepwise approach. During the first week, only lunch recipes are provided. In week 2, breakfast recipes are added. In week 3, dinner is added as well. For the other pillars—exercise, relaxation and sleep—"*menus*" and challenges are provided to get started and inspired, experience the impact of lifestyle changes, and integrate this into their daily life in their own way. Self-management of the participants' own health is the most important principle throughout the program.

References:

1. Pot GK, Battjes-Fries MC, Patijn ON, van der Zijl N, Pijl H, Voshol P. Lifestyle medicine for type 2 diabetes: practice-based evidence for long-term efficacy of a multicomponent lifestyle intervention (Reverse Diabetes2 Now). BMJ Nutr Prev Health. 2020 Aug 18;3(2):188-195. doi: 10.1136/bmjnph-2020-000081. PMID: 33521528; PMCID: PMC7841830.
2. Vries HD, Mudde AN. Predicting stage transitions for smoking cessation applying the attitude-social influence-efficacy model. Psychology & Health. 1998 13(2): 369-385. doi.org/10.1080/08870449808406757.
